# Metabolic reprogramming and epigenetic changes of vital organs in SARS-CoV-2–induced systemic toxicity

**DOI:** 10.1172/jci.insight.145027

**Published:** 2021-01-25

**Authors:** Shen Li, Feiyang Ma, Tomohiro Yokota, Gustavo Garcia, Amelia Palermo, Yijie Wang, Colin Farrell, Yu-Chen Wang, Rimao Wu, Zhiqiang Zhou, Calvin Pan, Marco Morselli, Michael A. Teitell, Sergey Ryazantsev, Gregory A. Fishbein, Johanna ten Hoeve, Valerie A. Arboleda, Joshua Bloom, Barbara Dillon, Matteo Pellegrini, Aldons J. Lusis, Thomas G. Graeber, Vaithilingaraja Arumugaswami, Arjun Deb

**Affiliations:** 1 Division of Cardiology, Department of Medicine, David Geffen School of Medicine,; 2 UCLA Cardiovascular Research Theme, David Geffen School of Medicine,; 3 Department of Molecular, Cell and Developmental Biology, Division of Life Sciences,; 4 Eli & Edythe Broad Center of Regenerative Medicine and Stem Cell Research,; 5 Molecular Biology Institute,; 6 California Nanosystems Institute,; 7 Department of Molecular and Medical Pharmacology, David Geffen School of Medicine,; 8 UCLA Metabolomics Center,; 9 Crump Institute for Molecular Imaging,; 10 Department of Human Genetics, David Geffen School of Medicine,; 11 Department of Pathology and Laboratory Medicine, David Geffen School of Medicine,; 12 Department of Biological Chemistry, David Geffen School of Medicine,; 13 Howard Hughes Medical Institute, and; 14 Department of Environment, Health and Safety, UCLA, Los Angeles, California, USA.

**Keywords:** COVID-19, Metabolism, Epigenetics, Intermediary metabolism

## Abstract

Extrapulmonary manifestations of COVID-19 are associated with a much higher mortality rate than pulmonary manifestations. However, little is known about the pathogenesis of systemic complications of COVID-19. Here, we create a murine model of SARS-CoV-2–induced severe systemic toxicity and multiorgan involvement by expressing the human ACE2 transgene in multiple tissues via viral delivery, followed by systemic administration of SARS-CoV-2. The animals develop a profound phenotype within 7 days with severe weight loss, morbidity, and failure to thrive. We demonstrate that there is metabolic suppression of oxidative phosphorylation and the tricarboxylic acid (TCA) cycle in multiple organs with neutrophilia, lymphopenia, and splenic atrophy, mirroring human COVID-19 phenotypes. Animals had a significantly lower heart rate, and electron microscopy demonstrated myofibrillar disarray and myocardial edema, a common pathogenic cardiac phenotype in human COVID-19. We performed metabolomic profiling of peripheral blood and identified a panel of TCA cycle metabolites that served as biomarkers of depressed oxidative phosphorylation. Finally, we observed that SARS-CoV-2 induces epigenetic changes of DNA methylation, which affects expression of immune response genes and could, in part, contribute to COVID-19 pathogenesis. Our model suggests that SARS-CoV-2–induced metabolic reprogramming and epigenetic changes in internal organs could contribute to systemic toxicity and lethality in COVID-19.

## Introduction

SARS-CoV-2 is a respiratory pathogen, but it can cause significant issues in other organ systems (1). Almost every organ system from the heart, brain, vasculature, hematopoietic, liver, skin, and others have been reported to be affected by COVID-19 and have resulted in complications such as acute cardiac dysfunction, thrombosis, immune dysfunction, and metabolic complications (2–8). Clinical studies during this pandemic have demonstrated that, in individuals hospitalized with COVID-19, the presence of extrapulmonary organ involvement is a predictor of significantly increased morbidity and mortality (2, 4, 9). Multiple tissues express the ACE2 receptor (10) and direct viral cytotoxicity, dysregulated immune response, and systemic cytokine release, microcirculatory defects, and thrombosis have been postulated to contribute to systemic toxicity in COVID-19 (1). However, the pathogenesis of extrapulmonary involvement and ensuing systemic toxicity in COVID-19 is largely unclear but remains paramount to our understanding of the disease presentation of COVID-19.

## Results

### A murine model of SARS-CoV-2–induced systemic toxicity.

**Figure 1:**
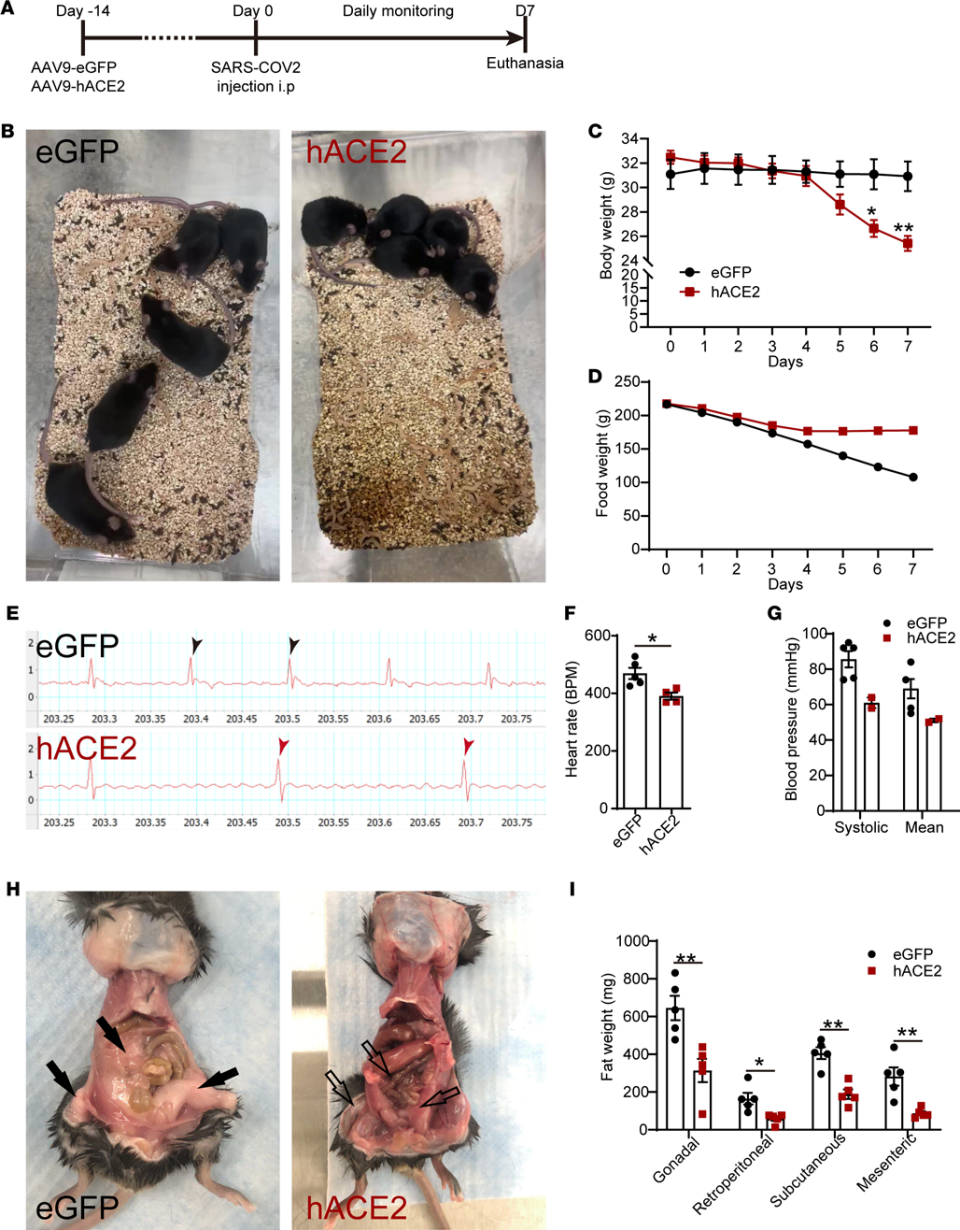
A murine model of SARS-CoV-2–induced systemic toxicity. (**A**) Temporal schematic of injection of hACE2-AAV-9 or eGFP-AAV-9 and SARS-CoV-2 injection. (**B**) Huddled posture of hACE2/SARS-CoV-2 mice compared with eGFP/SARS-CoV-2 controls. (**C**) Body weight of mice measured daily over 7 days (data shown as mean ± SEM, *n* = 5/group, *P < 0.05, **P < 0.01, 2-way ANOVA with Sidak’s multiple comparisons analysis). (**D**) Food consumption measured by assessing daily food weight/cage. (**E**) ECG strip demonstrating slower heart rate in hACE2/SARS-CoV-2 mice (arrows point to ventricular beats). (**F**) Resting heart rates (data shown as mean ± SEM, *n* = 5/group, *P < 0.05, Student’s *t* test, 2 tailed) and (**G**) blood pressure measured by tail cuff in anesthetized mice (data shown as mean ± SEM, *n* = 5/eGFP, *n* = 2/hACE2; 3/5 of hACE2 group had unrecordable blood pressures, Student’s *t* test, 2 tailed). (**H**) Ventral view at necropsy demonstrating changes in s.c., gonadal, and omental fat in eGFP and hACE2 SARS-CoV-2 groups (black and unfilled arrows). (**I**) Weight of fat dissected from different regions in eGFP and hACE2 SARS-CoV-2 groups (data shown as mean ± SEM, *n* = 5/group, *P < 0.05, **P < 0.01, Student’s *t* test, 2 tailed).

We generated a murine model of SARS-CoV-2–induced systemic toxicity by i.v. injecting WT male C57BL/6 mice (14–17 weeks) with an adeno associated virus serotype 9 (AAV-9) encoding the human ACE2 (hACE2) transgene and, 2 weeks later, injected SARS-CoV-2 virus (Isolate USA-WA1/2020) i.p. into these animals at a dose of 5 × 10^5^ TCID50/mouse (hACE2/SARS-CoV-2 group) (Figure 1A). We administered SARS-CoV-2 i.p., since we hypothesized that systemic rather than intranasal inoculation of SARS-CoV-2 would be more efficient in infecting multiple organs. AAV-9 is known to be distributed widely across internal organs after systemic delivery (11), and consistent with this, we confirmed hACE2 expression in multiple organs, including the heart, kidney, spleen, lung, liver, adipose tissue, and skeletal muscle of injected animals at 2 weeks following AAV-9 injection (Supplemental Figure 1A; supplemental material available online with this article; https://doi.org/10.1172/jci.insight.145027DS1). C57BL/6 animals of identical age and sex injected with eGFP-AAV-9 virus, but injected i.p. with an identical dose of SARS-CoV-2, served as controls (eGFP/SARS-CoV-2 group). Animals injected with the AAV-9 hACE2, but injected with vehicle rather than SARS-CoV-2, were used as an additional set of controls (hACE2/vehicle group). Within 7 days of SARS-CoV-2 injection, the animals harboring the hACE2 transgene (hACE2/SARS-CoV-2 group) demonstrated profound morbidity and severely restricted activity and were found huddled at the corner of the cage, in contrast to the normally active eGFP/SARS-CoV-2 animals (Figure 1B and Supplemental Video 1, A and B). The SWAB-seq method utilizing RNA sequencing (RNA-seq) as a readout for viral infection demonstrated robust amplification of viral genomes in hearts of hACE2/SARS-CoV-2 animals at 7 days after infection compared with eGFP/SARS-CoV-2 animals (12) (Supplemental Figure 1B). The hACE2/SARS-CoV-2 animals exhibited severe weight loss, wasting, and failure to thrive. An analysis of weight loss and food intake demonstrated approximately 22% ± 1% weight loss (mean ± SEM, *n* = 5/group, *P* < 0.01) within 7 days of SARS-CoV-2 injection, while the control eGFP/SARS-CoV-2 group did not exhibit any significant weight loss (*P* > 0.05) (Figure 1C). Food consumption was minimal and significantly lower in the hACE2/SARS-CoV-2 group compared with the eGFP/SARS-CoV-2 group, particularly from day 4 after SARS-CoV-2 virus injection. At the time of euthanasia (day 7), the hACE2/SARS-CoV-2 animals had consumed 67% less food in a week compared with the control eGFP/SARS-CoV-2 group (Figure 1D). Examination of the hACE2/vehicle-injected animals also did not demonstrate any morbidity, weight loss, or alteration in food consumption (Supplemental Figure 1, C–E). An electrocardiogram demonstrated bradycardia with a significant depression in resting heart rate (HR) of the hACE2/SARS-CoV-2 group compared with eGFP/SARS-CoV-2 group (resting HR of 390 beats/min in hACE2/SARS-CoV-2 [*n* = 5] versus 469 beats/min in eGFP/SARS-CoV-2 [*n* = 4]; mean ± SEM, *P* < 0.05) (Figure 1, E and F). Recording of blood pressure by noninvasive means demonstrated decreased systolic and mean arterial blood pressure in the hACE2/SARS-CoV-2 animals (with an unrecordable blood pressure in 3 of 5 animals in the hACE2/SARS-CoV-2 group), the bradycardia, and hypotension, consistent with extreme morbidity displayed by this group (Figure 1G). Since the hACE2/SARS-CoV-2 animals had lost approximately 25% of their body weight, had stopped consuming food, and appeared inactive and moribund, we had to euthanize the animals at day 7 after SARS-CoV-2 injection. At necropsy on gross examination, the hACE2/SARS-CoV-2 group demonstrated severe emaciation and loss of fat stores with significant reduction in gonadal, retroperitoneal, s.c., and mesenteric/omental fat compared with the eGFP/SARS-CoV-2 group (Figure 1, H and I). Taken together, these observations demonstrate a murine model of SARS-CoV-2–induced systemic toxicity with profound morbidity, wasting, weight loss, bradycardia, and failure to thrive within 7 days of SARS-CoV-2 injection.

### Multiple organ systems are affected in this murine model of SARS-CoV-2–induced systemic toxicity, mirroring human COVID-19 clinical phenotypes.

**Figure 2:**
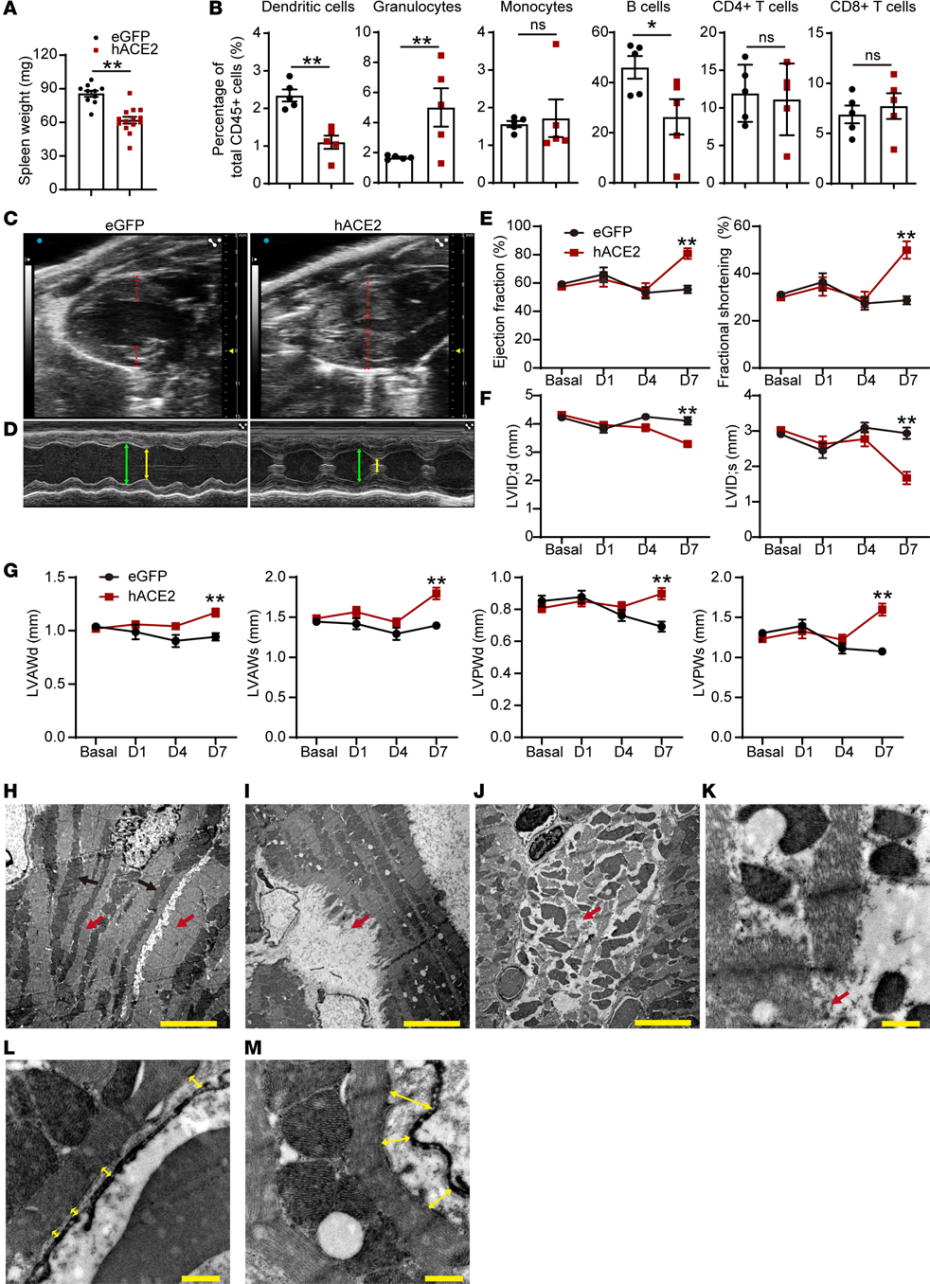
Involvement of major organ systems in a murine model of SARS-CoV-2–induced systemic toxicity. (**A** and **B**) Splenic weight (data shown as mean ± SEM; *n* = 10, eGFP; *n* = 14, hACE2; **P < 0.01, Student’s *t* test, 2 tailed) (**A**) and splenic cell counts of eGFP/SARS-CoV-2 and hACE2/SARS-CoV-2 animals (data shown as mean ± SEM; *n* = 5/group; *P < 0.05, **P < 0.01; Student’s *t* test, 2 tailed) (**B**). (**C** and **D**) B mode echocardiogram demonstrating thickness of heart walls (dotted lines) (**C**) and M mode echocardiogram demonstrating thickness and hyperdynamic contraction in diastole (green) and systole (yellow line) (**D**). (**E** and **F**) Ejection fraction and fractional shortening (**E**) and left ventricular internal diameter (LVID) in systole (s) and diastole (d) (**F**) (data shown as mean ± SEM; *n* = 5, eGFP; *n* = 9, hACE2; **P < 0.01, 2-way ANOVA with Sidak’s multiple comparisons analysis). (**G**) Thickness of the left ventricular anterior wall (LVAW) and posterior walls (LVPW) in systole (s) and diastole (d) between eGFP/SARS-CoV-2 and hACE2/SARS-CoV-2 animals (data shown as mean ± SEM, *n* = 5/eGFP, *n* = 9/hACE2, **P < 0.01, 2-way ANOVA with Sidak’s multiple comparisons analysis). (**H–M**) Transmission electron microscopy demonstrating cardiac muscle of eGFP/SARS-CoV-2 (**H**) hACE2/SARS-CoV-2 (**I** and **J**) animals showing parallel arrangement of myofibrils (red arrow) and mitochondria (black arrow) (**H**) and ‘ghost’ cell (**I**) with myocyte cell destruction (red arrow) (**J**) myofibrillar disarray with loss of parallel myofibrillar arrangement (red arrow) and (**K**) breaks in myofibrils seen with higher magnification (red arrow) (**L** and **M**). Transmission electron microscopy demonstrating myocyte to endothelial distance (yellow lines) in eGFP/SARS-CoV-2 (**L**) and hACE2/SARS-CoV-2 (**M**) heart, demonstrating wider intercellular gap. Scale bars: 5 μm (**H–J**) and 0.5 μm (**K–M**).

**Figure 3:**
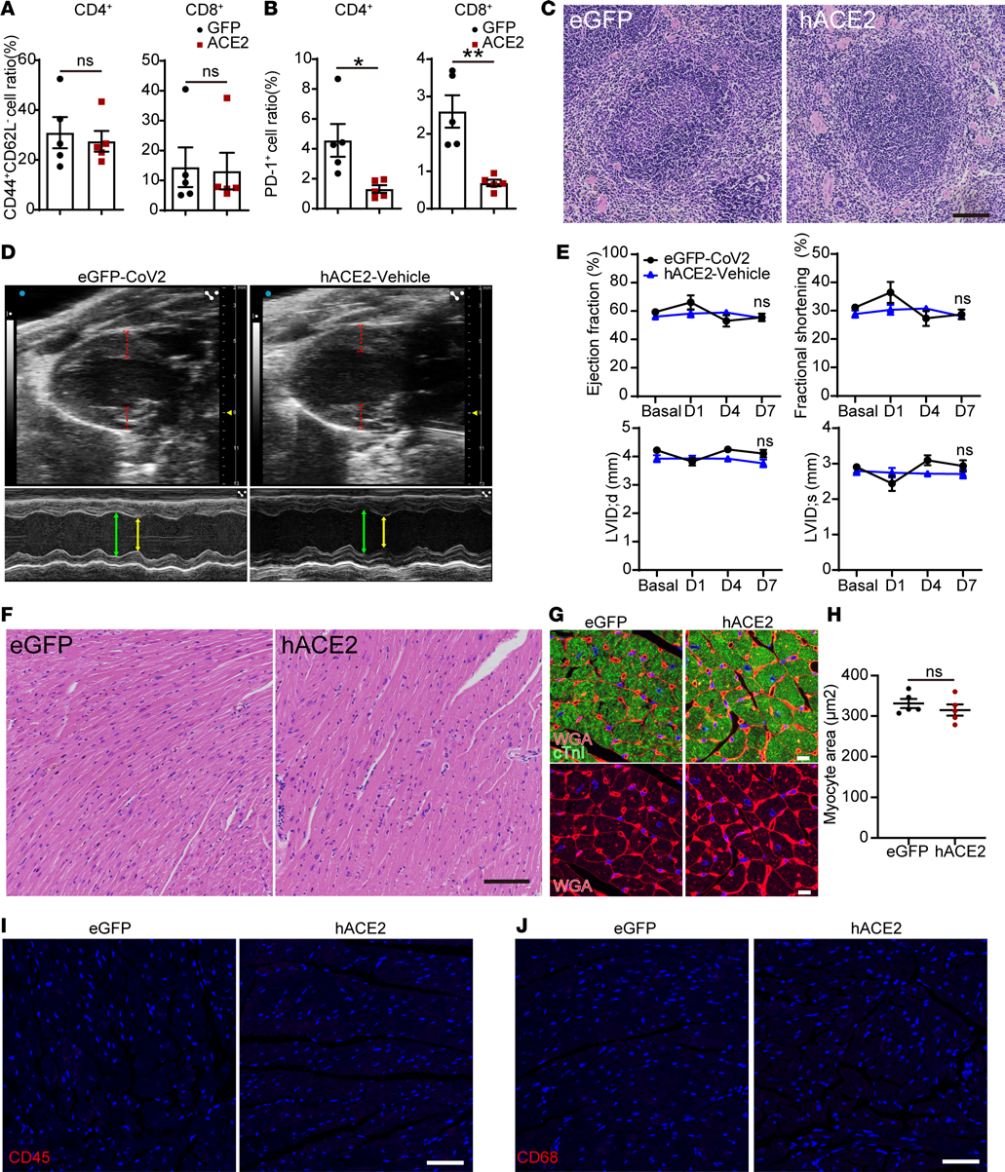
Extrapulmonary organ phenotypes in a murine model of SARS-CoV-2–induced systemic toxicity. (**A** and **B**) Analysis of splenic cells of hACE2/SARS-CoV-2 and eGFP/SARS-CoV-2 animals at 7 days following SARS-CoV-2 injection showing CD44^+^CD64L^–^ cells as a fraction of CD4^+^ and CD8^+^ T cells (**A**) and the fraction of CD4^+^ and CD8^+^ T cells expressing PD-1 (**B**) (data shown as mean ± SEM, *n* = 5/group, *P < 0.05, **P < 0.01, Student’s *t* test, 2 tailed). (**C**) H&E staining of the spleen of both groups. Scale bar: 100 μm. (**D**) B and M mode echocardiograms demonstrating no difference in thickness between eGFP/SARS-CoV-2 and hACE2/vehicle groups. (**E**) Ejection fraction, fractional shortening, and left ventricular internal dimension (LVID) in systole (s) and diastole (d) between eGFP/SARS-CoV-2 and hACE2/vehicle animals (data shown as mean ± SEM; *n* = 5, eGFP/SARS-CoV-2; *n* = 4, hACE2/vehicle; 2-way ANOVA with Sidak’s multiple comparisons analysis). (**F** and **G**) H&E staining of hearts (**F**) and immunostaining for cardiac troponin and wheat germ agglutinin (WGA) (**G**) to determine myocyte surface area between eGFP and hACE2/SARS-CoV-2 groups. Scale bars: 100 μm (**F**) and 10 μm (**G**). (**H**) Myocyte surface area in hearts of both groups (*n* = 5/group, Student’s *t* test, 2 tailed). (**I** and **J**) Immunostaining for CD45 (hematopoietic cell) (**I**) and CD68 (macrophage marker) (**J**) demonstrates lack of expression in the hearts of both groups (*n* = 5/group). Scale bar: 50 μm.

We next investigated how multiple organ systems are affected in the hACE2/SARS-CoV-2 animals. Complete blood counts demonstrated normal hemoglobin, RBC counts and associated RBC metrics, and normal total WBC counts, with no significant differences between the hACE2/SARS-CoV-2 and control eGFP/SARS-CoV-2 animals (Table 1). However, examination of the differential WBC counts demonstrated significant granulocytosis and lymphopenia in the hACE2/SARS-CoV-2 animals compared with the control eGFP/SARS-CoV-2 animals (Table 2). The hACE2/SARS-CoV-2 group demonstrated splenic atrophy with significant reduction in splenic weights (Figure 2A). Examination of the cellular composition of the spleen demonstrated significantly decreased DCs, as well as B lymphocytes, with significant increase in granulocytes, mirroring granulocytosis in peripheral blood (Figure 2B). There were no differences in the fraction of splenic monocytes and CD4^+^ or CD8^+^ T cells between the hACE2/SARS-CoV-2 group and the eGFP/SARS-CoV-2 group (Figure 2B). We next examined markers of immune activation. Both CD4^+^ and CD8^+^ T cells from the spleens of hACE2/SARS-CoV-2 and eGFP/SARS-CoV-2 groups displayed low percentages of CD44^+^CD62L^–^ cell subsets (T effector/effector memory, T_E/EM_) (Figure 3A). Further analysis demonstrated that both CD4^+^ and CD8^+^ T cells expressed low levels of Programmed Death-1 (PD-1), a major marker of T cell exhaustion, in the eGFP/SARS-CoV-2 group, and T cells from hACE2/SARS-CoV-2 had further reduced PD-1 expression (Figure 3B). These results indicate that T cells are not fully activated in either the hACE2/SARS-CoV-2 group or the eGFP/SARS-CoV-2 group following SARS-CoV-2 injection. Histological examination of the spleen did not demonstrate any abnormalities (Figure 3C**)**. Collectively, these observations of the hematopoietic system mirror reported human clinical phenotypes, with lymphopenia being a cardinal laboratory finding observed in almost 80% of hospitalized individuals with COVID-19 and associated with an adverse prognosis (3). Neutrophilia with an increased neutrophil/lymphocyte ratio and splenic atrophy in COVID-19 has been shown to be associated with adverse prognosis and significantly worse clinical outcomes (13). Suboptimal activation of CD4^+^ and CD8^+^ T cells, as observed by us in this model, is consistent with recent reports of diverse patterns of T cell activation in humans with COVID-19 (14) and suboptimal or inappropriate T cell responses being associated with severe disease (15).

Cardiac involvement in individuals with COVID-19 has been demonstrated in multiple clinical studies to be associated with a significantly higher mortality rate (4). Imaging/autopsy studies have demonstrated pathologic features such as myocardial edema, altered cardiac function, and inflammation (2). Since AAV9 exhibits robust tropism for the heart, and the experimental animals had evidence of depressed HR, we examined the animals for cardiac involvement. We first examined cardiac function by performing echocardiography and observed that the hearts of these animals exhibited significant wall thickening of 25%–30% by day 7 compared with control eGFP/SARS-CoV-2 animals (Figure 2, C and G). Examination of hearts of hACE2/vehicle-injected animals at identical time points also did not reveal any thickening of walls or hyperdynamic function (Figure 3D). The cardiac function of the hACE2/SARS-CoV-2 animals was hyperdynamic compared with the eGFP/SARS-CoV-2 animals (Figure 2D), with significantly increased ejection fraction and fractional shortening (Figure 2E), and this was associated with a significant reduction in ventricular chamber size (Figure 2F). No such changes were observed in the hACE2/vehicle groups (Figure 3E). Wall thickening observed on echocardiography can occur from cardiac hypertrophy or myocardial edema. To distinguish between these, we examined heart sections, but there was no histological evidence of myocyte hypertrophy (Figure 3, F–H), thereby suggestive of myocardial edema as a potential cause of wall thickening. There was also no evidence of increased inflammatory infiltrate in the hearts of hACE2/SARS-CoV-2 animals compared with the eGFP/SARS-CoV-2 group (Figure 3, I and J). The hyperdynamic contraction of the cardiac chambers is reminiscent of cardiac changes observed in early systemic inflammatory response syndrome (SIRS) or sepsis in humans with decreased afterload. The lack of inflammation in the heart is also consistent with recent autopsy reports in humans with COVID-19 who did not demonstrate any significant inflammatory infiltrate in the hearts of individuals infected with SARS-CoV-2 (16). Myocardial edema has been reported to occur in hearts of humans with COVID-19 and has been thought to be pathogenic (17). We examined ultrastructural features of the cardiomyocyte to determine abnormalities that may not be obvious on histology. Transmission electron microscopy demonstrated numerous myocyte “ghost” cells, with myocyte cell death, and extreme myofibrillar disarray and myofibrillar breaks in the hearts of hACE2/SARS-CoV-2 but not in eGFP/SARS-CoV-2 animals (Figure 2, H–K). Interstitial edema would increase the intercellular distance between a myocyte and a nonmyocyte cell. To determine the presence of myocardial edema on electron microscopy, we examined the intercellular distance between the cardiac muscle cell and the neighboring endothelial cell in the cardiac interstitium and found the intercellular distance to be significantly greater by 3 fold in the hACE2/SARS-CoV-2 animals, suggestive of myocardial edema (0.26 ± 0.04 μm in eGFP/SARS-CoV-2 group versus 0.80 ± 0.25 μm in hACE2/SARS-CoV-2 animals, *P* < 0.05) (Figure 2, L and M). Collectively, these observations demonstrating the presence of myocyte cell death, myofibrillar disarray, myocardial edema, thickened ventricular walls, and depressed HR recapitulate, in part, clinical phenotypes of cardiac involvement in COVID-19. The presence of myofibrillar disarray and ghost cells in the myocardium on electron microscopy are consistent with and can lead to cardiac arrhythmias reported in individuals with COVID-19.

We next performed histological examination of the kidneys, liver, lung, and adipose tissue to determine any SARS-CoV-2–induced pathological changes in those organs. On H&E staining, the architecture of the kidneys appeared to be grossly normal, without any inflammation or pathological changes (Supplemental Figure 2A). Serum biochemistry demonstrated that serum sodium, potassium, and creatinine were within the normal range and similar to the hACE2/vehicle animals (Table 3). The serum blood urea nitrogen (BUN) was significantly higher in the hACE2/SARS-CoV-2 animals, which likely reflects mild renal impairment or dehydration from inadequate fluid intake (Table 3). Since the hACE2 gene can potentially affect sodium/potassium homeostasis, hACE2/vehicle animals were used as controls for these biochemical measurements. The liver and lung did not show any abnormality on histological examination (Supplemental Figure 2, B and C), and serum liver transaminases (alanine transaminase) were not elevated, thus excluding any acute liver injury (Table 3). There were no histological abnormalities or differences in the skeletal muscle between the 2 groups (Supplemental Figure 2D), and small intestine and large intestine histology was also normal (Supplemental Figure 2, E and F). As the animals exhibited severe wasting and reduction in fat stores, we subjected fat tissue to histological examination and observed significant reduction in size of adipocytes in the hACE2/SARS-CoV-2 animals compared with control eGFP/SARS-CoV-2 animals (Supplemental Figure 2G).

### SARS-CoV-2 alters expression of a multitude of genes regulating key metabolic pathways in vital organs.

**Figure 4:**
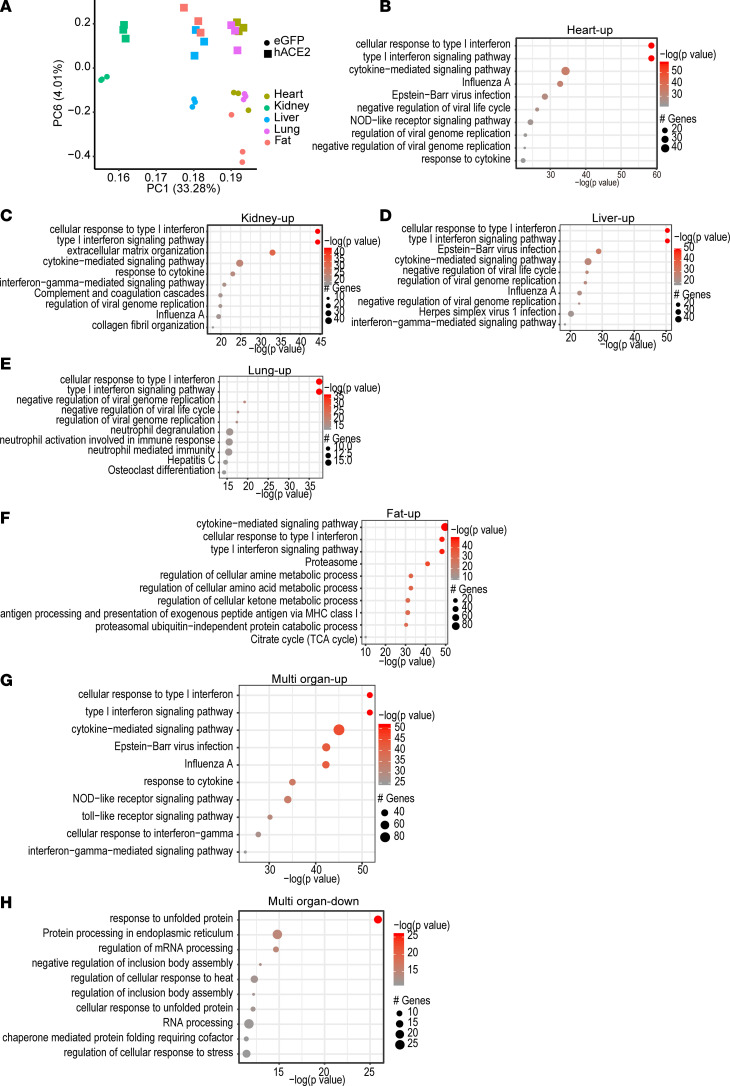
Pattern of gene expression changes in multiple organs in hACE2/SARS-CoV-2 animals compared with eGFP/SARS-CoV-2 animals at 3 days after SARS-CoV-2 infection. (**A**) Principal component (PC) analysis of differentially expressed genes in multiple organs shows separation of organs according to viral infection by PC6 (*n* = 3/group). (**B**–F) GO analysis demonstrates pathways enriched by differentially upregulated genes in the heart (**B**), kidney (**C**), liver (**D**), lung (**E**), and adipose tissue (**F**). (**G** and **H**) Pathways enriched by differentially upregulated genes that are common to the above organs (**G**) and pathways enriched by differentially downregulated genes that are common to the above organs (**H**).

As the hACE2/SARS-CoV-2 animals exhibited rapidly developing marked systemic toxicity, we performed analysis of gene expression changes in vital organs to obtain insight into how pathophysiologic changes occurring in each organ contributed to systemic toxicity. As the animals started to lose weight and develop systemic toxicity from day 4 onward, we harvested internal organs first at day 3 after SARS-CoV-2 injection and performed bulk RNA-seq to determine early changes in gene expression (Supplemental Table 1). Principal component (PC) analysis demonstrated that PC6 clearly separated virus-infected tissues (hACE2/SARS-CoV-2) from those in control eGFP/SARS-CoV-2 animals (Figure 4A). Gene ontology (GO) of significantly upregulated genes (adjusted *P* value [*P*_adj_] < 0.05) demonstrated that the pathways related to type I IFN signaling, cellular response to IFN, and cytokine-mediated signaling pathways were primarily enriched in multiple organs, including the heart, kidney, lung, liver, and adipose tissue, compared with those of eGFP/SARS-CoV-2 animals (Figure 4, B–F). Genes regulating canonical IFN signaling, such as the IFN regulatory factors (IRF) family of transcription factors, RNA-specific adenosine deaminase (ADAR), and the IFN-induced transmembrane (IFITM) family of genes — which resist viral cell entry — were upregulated in all organs examined (Figure 4G and Supplemental Table 1). GO analysis of genes that were differentially downregulated in multiple organs demonstrated enrichment of pathways related to endoplasmic reticulum stress, unfolded protein cell response, and spliceosome-mediated mRNA processing (Figure 4H). Taken together, these data demonstrate an antiviral immune response being generated in multiple organs in the hACE2/SARS-CoV-2 animals compared with control eGFP/SARS-CoV-2 animals within 3 days of SARS-CoV-2 injection.

**Figure 5:**
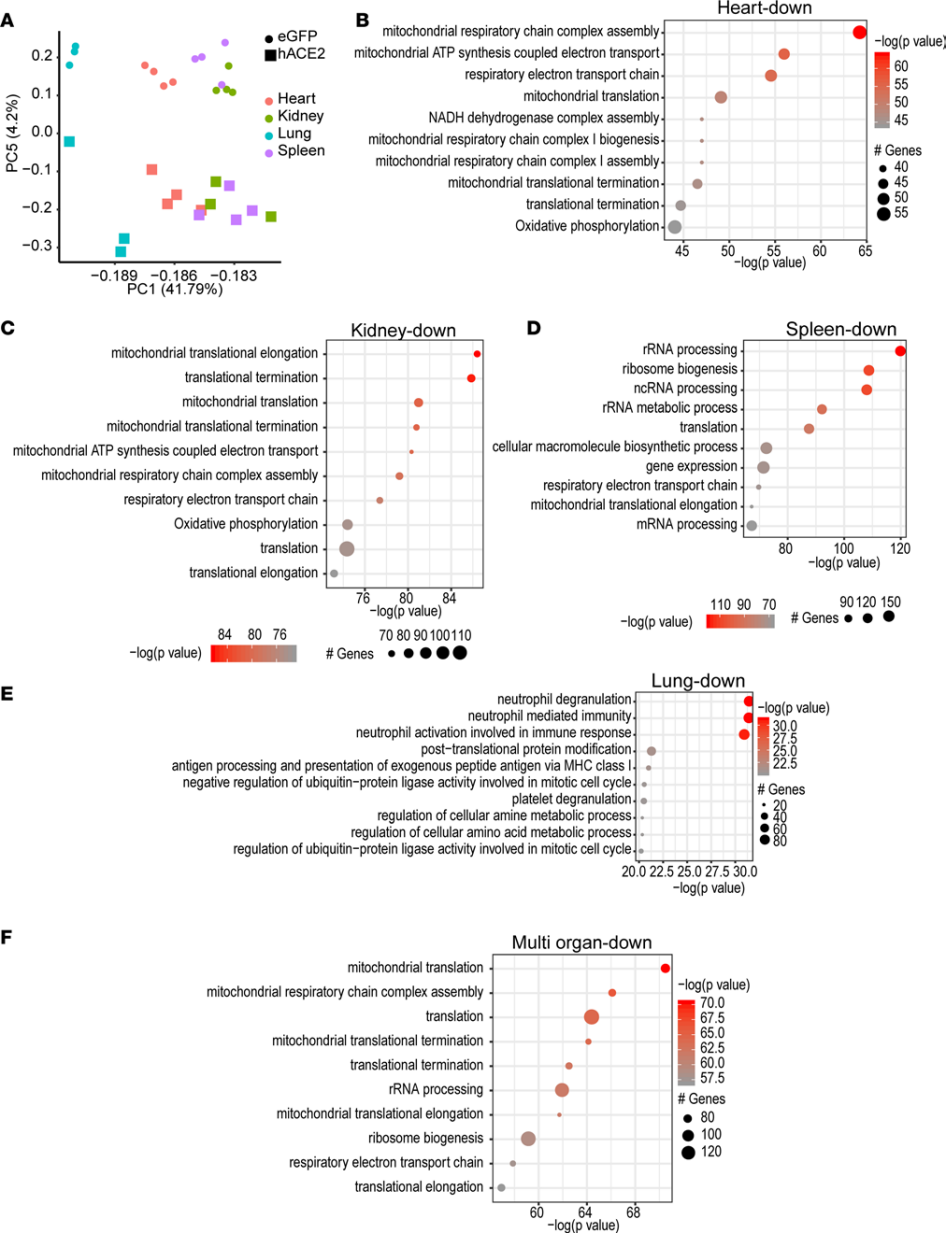
Systemic toxicity in SARS-CoV-2 is associated with downregulation in expression of genes regulating oxidative phosphorylation and TCA cycle in multiple organs. (**A**) Principal component (PC) analysis of differentially expressed genes in multiple organs shows separation of organs according to viral infection by PC5. Heart: *n* = 4, eGFP; *n* = 4, hACE2. Kidney: *n* = 4, eGFP; *n* = 3, hACE2. Lung: *n* = 3, eGFP; *n* = 3, hACE2. Kidney: *n* = 4, eGFP; *n* = 4, hACE2. (**B–E**) GO analysis demonstrating major pathways (ranked by *P* value) depressed in heart (**B**), kidney (**C**), spleen (**D**), and lung (**E**) in hACE2/SARS-CoV-2 animals. (**F**) GO analysis showing major pathways enriched by downregulated genes across multiple organs in hACE2/SARS-CoV-2 animals.

As the animals were severely moribund and had to be euthanized at 7 days after SARS-CoV-2 injection, we next harvested internal organs at day 7 and determined gene expression changes by performing bulk RNA-seq to obtain insight into temporal changes in gene expression that could contribute to pathogenesis of severe systemic toxicity (Supplemental Table 2). eGFP/SARS-CoV-2 animals harvested at an identical time points served as controls. PC analysis of differentially expressed genes demonstrated that PC5 clearly separated gene expression changes in major organs of hACE2/SARS-CoV-2 animals compared with eGFP/SARS-CoV-2 (Figure 5A). We examined all genes that were significantly differentially expressed (*P* < 0.05) in the heart, kidney, spleen, and lung. GO analysis of significantly downregulated genes in hearts of hACE2/SARS-CoV-2 animals compared with the eGFP/SARS-CoV-2 group demonstrated inhibition of mitochondrial electron transport chain, including biogenesis and assembly of the electron transport complexes and oxidative phosphorylation (Figure 5B). Similarly, downregulated genes in the kidney affected various aspects of mitochondrial function, including mitochondrial translation and elongation, electron transport chain, electron transport chain assembly, and mitochondrial ATP synthesis (Figure 5C). Downregulated genes in spleens of hACE2/SARS-CoV-2 animals were enriched in rRNA processing and ribosomal function, as well as in the mitochondrial electron transport chain, mitochondrial translation, elongation pathways, and oxidative phosphorylation (Figure 5D). Downregulated genes in the lung affected immune response pathways related to neutrophil-mediated immunity and activation, MHC-mediated antigen processing, and also oxidative phosphorylation (Figure 5E).

**Figure 6:**
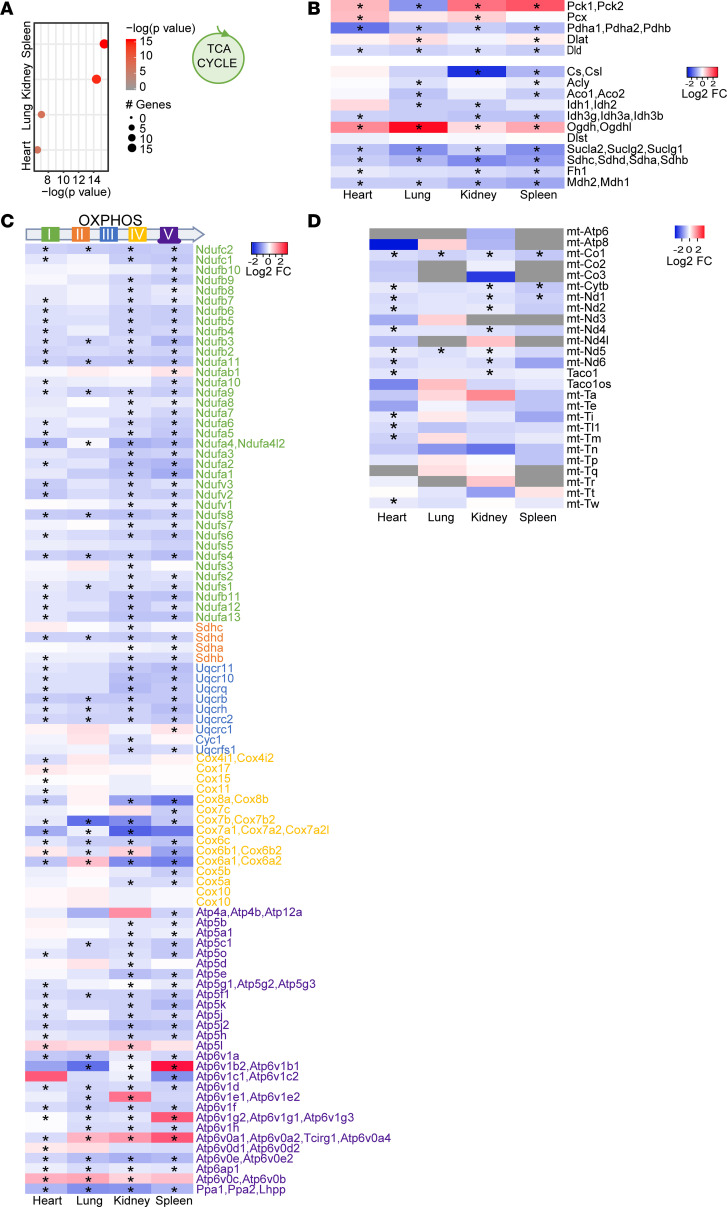
Systemic toxicity in SARS-CoV-2 is associated with changes in expression of specific metabolic genes of the TCA cycle or oxidative phosphorylation. (**A**) Downregulation of TCA cycle genes in multiple organs ranked according to the *P* value. (**B**) Heatmap showing gene expression changes of specific TCA cycle genes that are downregulated in multiple organs (*P_adj_ < 0.05). (**C**) Heatmap showing expression changes of genes related to electron transport chain (color coded) in multiple organs to demonstrate its relationship to specific complexes in the electron transport chain (*P_adj_ < 0.05). (**D**) mtDNA-encoded oxidative phosphorylation genes that are significantly downregulated in the heart and other organs (*P_adj_ < 0.05). Wald test statistic was used to obtain *P* values, further corrected for FDR with Benjamini-Hochberg procedure.

GO analysis of upregulated genes demonstrated that the predominant signaling pathways affected in the heart, kidney, lung, and spleen were related to regulation of transcription, mRNA biogenesis, splicing, and processing (Supplemental Figure 3A). Strikingly, we found that SARS-CoV-2 induced similar patterns of gene expression changes, regardless of the type of organ affected. We performed differential expression analysis between hACE2/SARS-CoV-2 and eGFP/SARS-CoV-2 animals across heart, kidney, lung, and spleen with a model that considers the different organs as variables. We plotted the significantly up- and downregulated genes and observed similar gene expression pattern changes in these organs (Supplemental Figure 3B). We performed GO for the genes commonly downregulated in multiple organs of hACE2/SARS-CoV-2 animals and observed significant enrichment for pathways related to oxidative phosphorylation and electron transport chain (Figure 5F). As the TCA cycle is intimately connected to the electron transport chain, we next examined the differential expression of genes regulating various steps of the TCA cycle across 4 organs and observed significant downregulation of TCA cycle genes in all organs examined (Figure 6A). We examined all differentially expressed genes of the TCA cycle and observed that genes regulating multiple steps of the TCA cycle were downregulated across the heart, lung, kidney, and spleen (Figure 6B). Next, we analyzed genes related to oxidative phosphorylation that were differentially expressed and observed that, across multiple organs, there was a consistent pattern in significantly decreased expression of genes encoding for various subunits of electron transport chain complexes driving oxidative phosphorylation (Figure 6C). Various subunits of the electron transport chain complexes are encoded both by mitochondrial and nuclear DNA. Since the heart has abundant mitochondria, we examined the expression of mtDNA-encoded genes and observed significantly decreased expression of mitochondrial genes in hearts as well as other organs of hACE2/SARS-CoV-2 animals compared with eGFP/SARS-CoV-2 animals, demonstrating an effect of SARS-CoV-2 in depressing transcription of both nuclear and mtDNA-related metabolic genes (Figure 6D). We examined mitochondria with phosphotungstic acid hematoxylin stain (PTAH), but we did not see any obvious changes in mitochondrial numbers in the kidneys and heart between hACE2/SARS-CoV-2 and eGFP/SARS-CoV-2 animals (Supplemental Figure 4, A–D). Electron microscopy of the heart also did not demonstrate any obvious ultrastructural defects in the mitochondria between the 2 groups (Supplemental Figure 4, E and F). We also compared gene expression changes in all organs at 3 days versus 7 days following SARS-CoV-2 injection and, from a transcriptome-wide analysis, found the gene expression changes to be highly distinct, with little correlation between these time points (Supplemental Figure 3C). Oxidative phosphorylation genes were not affected at day 3, while the prominent IFN immune response seen at day 3 was no longer evident at day 7, though a reduced IFN subset was present at both time points (Supplemental Figure 3, D and E). Disruption of circadian clock genes were observed at both time points, and previous studies have suggested clock disruption to aid viral pathogenesis (Supplemental Figure 3F) (18). Collectively, these data suggest the pathogenesis of the disease is associated with distinct temporal transcriptional patterns that strongly correlate with disease progression and development of morbidity in these animals. Our observations demonstrate that, within 7 days of infection, morbidity, wasting, and failure to thrive in these animals is associated with significant decrease in expression of genes related to key cellular metabolic processes regulating aerobic cellular respiration and energy production.

### hACE2/SARS-CoV-2 animals exhibit decreased TCA cycle metabolites in serum and altered DNA methylation patterns in vital organs.

**Figure 7:**
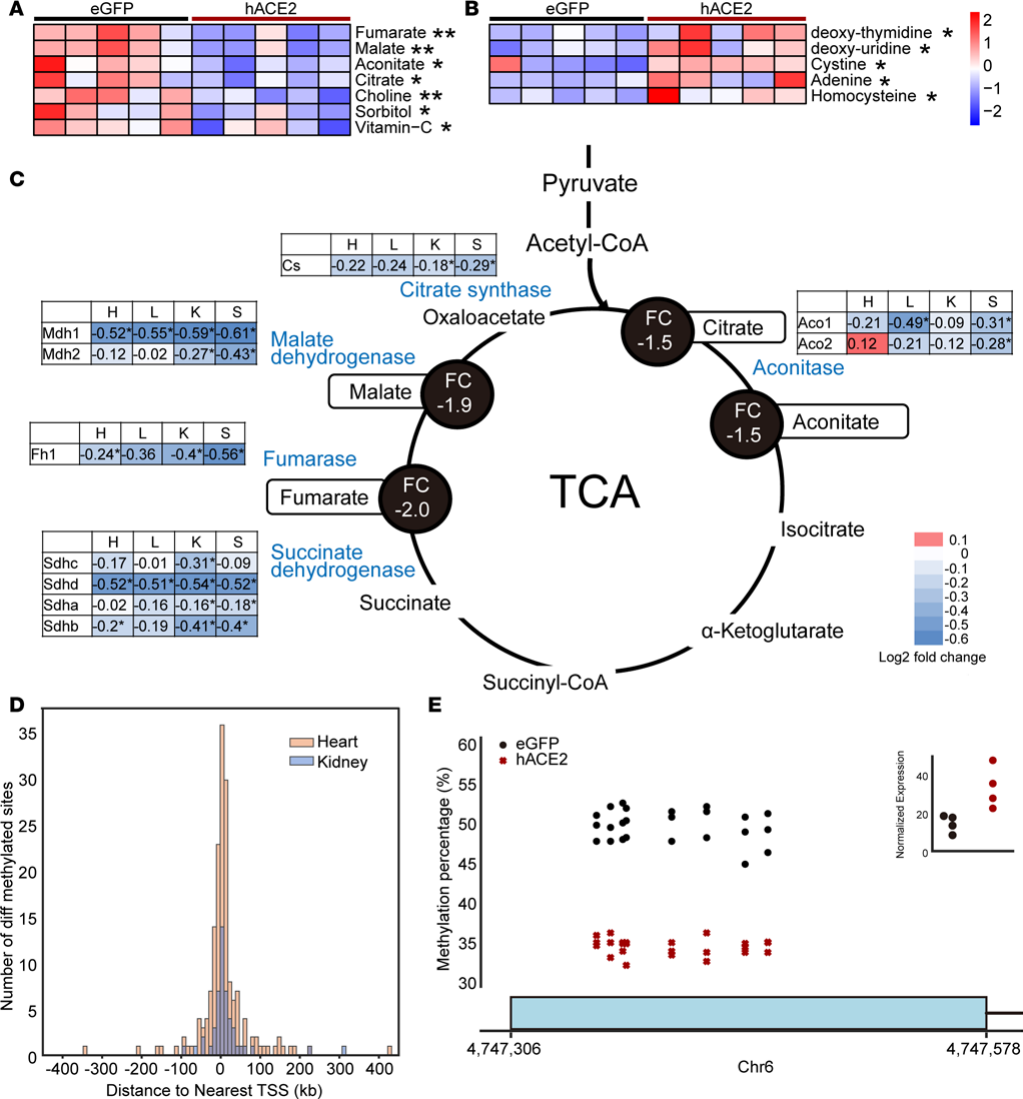
SARS-CoV-2–induced systemic toxicity is associated with decreased serum TCA cycle metabolites and DNA methylation changes in vital organs. (**A** and **B**) LC/MS demonstrating significantly decreased (**A**) and significantly increased (**B**) metabolites in serum of hACE2/SARS-CoV-2 animals compared with control eGFP/SARS-CoV-2 animals (data shown as mean ± SEM, *n* = 5/group, **P < 0.01, Student’s *t* test, 2 tailed). (**C**) Downregulated TCA cycle genes in multiple organs (heart [H], lungs [L], kidney [K], spleen [S]) of hACE2/SARS-CoV-2 animals compared with eGFP/SARS-CoV-2 animals (*P < 0.05). Significantly downregulated genes are shown in blue font with accompanying log_2_ fold changes across multiple organs. Metabolites generated by enzymes encoded by affected genes are highlighted, and serum fold changes are shown in black circles. (**D**) Histogram showing the number of differentially methylated sites plotted as a distance from a differentially methylated site to the nearest transcription start site (TSS) (5kb bins) for both heart (median distance = 2424 bp) and kidney (median distance = 6390 bp) tissue (*n* = 3/group). (**E**) Percentage of CpG methylated sites spanning the first exon of Peg10 gene in hearts of hACE2/SARS-CoV-2 animals and associated change in gene expression (red) and eGFP/SARS-CoV-2 animals (black) (*n* = 3, *q* < 0.01). Sliding Linear Model method was used to estimate *q* values.

Our data so far suggest that SARS-CoV-2 depresses the expression of genes regulating critical components of oxidative phosphorylation and TCA cycle in multiple organs. Furthermore, defects in the electron transport chain are known to depress the TCA cycle secondary to accumulation of NADH. A potential depressive effect on oxidative phosphorylation and TCA cycle would have profound consequences on cellular physiology and metabolism, especially of highly metabolic organs. We hypothesized that a depressive effect of the electron transport chain and TCA cycle in multiple organs would be reflected in decreased levels of TCA cycle metabolites in peripheral blood. To address this question, we performed metabolomic profiling of serum collected from hACE2/SARS-CoV-2 and eGFP/SARS-CoV-2 animals by liquid chromatography–mass spectrometry (LC/MS) at 7 days following SARS-CoV-2 injection. We observed that the TCA cycle metabolites citrate, fumarate, malate, and aconitate were significantly lower in the hACE2/SARS-CoV-2 group compared with the control group (Figure 7A). These observations provide potential biochemical evidence that, consistent with organ-wide transcriptional downregulation of multiple genes affecting the electron transport chain and TCA cycle, TCA cycle and oxidative phosphorylation are likely functionally depressed in vivo. Choline levels were also significantly decreased in hACE2/SARS-CoV-2 animals (Figure 7A), and choline and choline derivatives have been recently reported to be downregulated in patients with severe manifestations of COVID-19 (19) and are thought to be secondary to increased choline uptake by macrophages during polarization (20). Metabolites that were upregulated in the serum of hACE2/SARS-CoV-2 animals included deoxy-thymidine, deoxy-uridine, adenine, cystine, and homocysteine (Figure 7B). Although the physiologic significance of circulating adenine or deoxyuridine is not understood, elevated homocysteine is associated with endothelial damage in humans (21).

To provide corroborative evidence of the effects of SARS-CoV-2 in regulating cellular metabolic pathways, we infected human pluripotent stem cell–derived (hPSC-derived) cardiac muscle cells in vitro with SARS-CoV-2. Within 48 hours of infection, most human cardiac muscle cells underwent cell death (Supplemental Figure 5, A and B). Analysis of gene expression changes at 24 and 48 hours demonstrated that significantly downregulated genes related to electron transport chain and oxidative phosphorylation (Supplemental Figure 5, C and D, and Supplemental Table 3). We next examined the set of genes that were downregulated in both the hPSC-derived cardiomyocytes after SARS-CoV-2 infection and in the hearts of hACE2/SARS-CoV-2 animals at day 7 following SARS-CoV-2 infection. GO analysis of this common set of downregulated genes again demonstrated mitochondrial electron transport chain and oxidative phosphorylation pathways to be primarily affected (Supplemental Figure 5E). Finally, we examined the relationship between the decreased levels of serum metabolites and downregulated genes affecting multiple steps in the TCA cycle across multiple organs. We observed that decreased expression of genes regulating specific steps in the TCA cycle across all 4 tissues examined was generally associated with decreased levels of the serum metabolite generated by the enzyme encoded by the gene, thus demonstrating a high degree of concordance between tissue gene expression changes and changes in serum metabolites (Figure 7C). In particular, genes encoding for the TCA cycle enzymes succinate dehydrogenase, fumarase, malate dehydrogenase, citrate synthase, and aconitase exhibited decreased expression in multiple tissues in hACE2/SARS-CoV-2 animals compared with eGFP/SARS-CoV-2 animals, with a corresponding significant decrease in serum fumarate, malate, citrate, and aconitate, which represent TCA cycle metabolites generated by the enzymes (Figure 7C). A depression in TCA cycle would also affect fatty acid β oxidation as a source of acetyl CoA, and we hypothesized that this may lead to abnormalities of intracellular lipid homeostasis. We performed Oil Red O staining and observed robust adipose tissue deposition in the heart, kidney, and liver of hACE2/SARS-CoV-2 animals but not in control eGFP/SARS-CoV-2 group (Supplemental Figure 5, F–H). The peripheral utilization of adipose tissue, likely secondary to caloric restriction with visceral loading of fat, is consistent with a model of functional depression of oxidative phosphorylation in multiple tissues.

Given the profound and rapid changes in gene expression in multiple tissues, we next investigated whether epigenetic changes could potentially contribute to changes in gene expression in multiple organs. For this purpose, we performed DNA methylation analysis of the heart and kidney at 7 days following SARS-CoV-2 injection in the hACE2/SARS-CoV-2 and eGFP/SARS-CoV-2 animals. We identified 172 differentially methylated sites in the heart and 49 in the kidney (*q* < 0.01) between the hACE2/SARS-CoV-2 and eGFP/SARS-CoV-2 animals that were distributed across the host cell genome (Supplemental Figure 6, A and B, and Supplemental Table 4). A histogram showing the distance from a differentially methylated site to the nearest transcription start site (TSS) demonstrated a median distance of 2424 (bp) for the heart and 6390 bp for the kidney (Figure 7D). The number of differentially methylated sites was much greater in the heart than in the kidney, indicating tissue-specific methylation responses to SARS-CoV-2 (Figure 7D). The nearest gene to 26 heart and 3 kidney differentially methylated sites overlapped with the differentially expressed genes. Of those, 2 genes were associated with multiple differentially methylated sites in heart, paternally expressed 10 (*Peg10*; *n* = 7) and *Ece1* (*n* = 2). Particularly for Peg10, the differentially methylated sites were located within the first exon, and the hypomethylation pattern was consistent with higher expression of Peg10 in hearts of hACE2/SARS-CoV-2 animals compared with eGFP/SARS-CoV-2 animals (Figure 7E). Peg10 is a retrotransposon-derived paternally expressed imprinted gene, and loss of function of this gene results in early embryonic lethality (22, 23). Peg10 has been observed to regulate cell proliferation and has been postulated to bind to viral transcription regulators of the Ebola virus, potentially affecting viral replication and effects on tissues (24, 25). The differential methylated regions for *Ece1* (endothelin converting enzyme-1) were 13,000 bp upstream of the TSS, and increased methylation in the hACE2/SARS-CoV-2 was associated with altered gene expression (Supplemental Figure 6C). Ece1 is known to regulate proteolysis of endothelin precursors into biologically active peptides (26), and loss of function is associated with cardiac defects, generalized edema, and autonomic dysfunction (27). Although the physiological significance of SARS-CoV-2–altered DNA methylation patterns is not clear from our study, our model provides proof of concept that such epigenetic changes do occur soon after SARS-CoV-2 infection and can potentially lead to persistent transcriptional changes affecting tissue homeostasis and organ function.

## Discussion

We demonstrate a murine model of severe SARS-CoV-2 systemic toxicity that can be generated easily by first administering the hACE2 transgene by AAV-9 delivery, followed 2 weeks later with i.p. administration of SARS-CoV-2. Previous reports with nasal inoculation of the virus did not demonstrate severe morbidity, likely related to a higher systemic viral burden with i.p. versus intranasal delivery (28, 29). Our model recapitulates many commonly reported clinical phenotypes of humans with COVID-19, including splenic atrophy, lymphopenia with decreased B cell counts, granulocytosis, cardiac involvement with myofibrillar disarray, myocyte loss, myocardial edema, and bradycardia. The outcomes of systemic toxicity or morbidity can be easily measured in our model by determining weight loss, food intake, and activity throughout the 7 days following SARS-CoV-2 injection. The rapid development of morbidity and near organismal demise makes our model appealing for testing therapeutic agents such as antiviral drugs or vaccines. One limitation in our model is the i.p. route of SARS-CoV-2 infection, which is not a natural route of infection for humans. However, systemic viremia has been noted and implicated in multiorgan involvement in humans with COVID-19 (30), and this remains the primary reason we hypothesized that systemic injection of the virus would facilitate multiorgan seeding and systemic complications; therefore, this is the rationale for the i.p. delivery. Despite severe systemic symptoms that developed within 7 days, we did not see any evidence of persistent inflammation on histological examination of tissues. These findings are consistent with recent clinical reports that have also noted absence of circulating inflammatory cytokines in humans critically ill with COVID-19 (31). However, the transcriptional changes noted in different organs, particularly downregulation of genes regulating oxidative phosphorylation associated with decreased levels of specific TCA cycle metabolites and abnormalities in lipid homeostasis, suggests that functional suppression of mitochondrial function and aerobic cellular respiration may contribute to systemic toxicity. Differential expression of genes regulating metabolism, TCA cycle, and oxidative phosphorylation have been noted following SARS-CoV-2 infection of human cell lines, and metabolic reprogramming of monocytes under high-glucose conditions in vitro is thought to promote SARS-CoV-2 replication (32, 33). Defects in oxidative phosphorylation in humans commonly lead to lactic acidosis, but lactate levels were not elevated in the hACE2/SARS-CoV-2 animals. Increased serum lactate would be dependent on the degree of depression of oxidative phosphorylation in multiple tissues. Tissue variations in the degree of viral infection and, hence, depression in oxidative phosphorylation — coupled with decreased activity of the animals — could have attenuated a significant increase in serum lactate levels. In this regard, only 30% of children with disorders of the electron transport chain have elevated venous lactate, demonstrating the complexities of electron transport chain defects and presence of lactacidosis (34).

Our experiments with infection of human cardiac muscle cells with SARS-CoV-2 support our observations of a direct effect of the virus in depressing expression of genes regulating oxidative phosphorylation and electron transport chain. Significantly decreased levels of TCA metabolites (citrate, malate, fumarate, aconitate) in peripheral blood could potentially serve as biochemical markers of altered cellular metabolism following SARS-CoV-2 infection. In this regard, a recent report demonstrates that decreased citrate levels in peripheral blood of individuals with COVID-19 is associated with a worse prognosis (35). The prognostic value of circulating levels of malate, fumarate, and aconitate or other TCA metabolites in humans with COVID-19 currently remains unclear, but TCA cycle metabolites are known to exert nonmetabolic signaling effects regulating innate and adaptive immunity, lipid and nucleotide synthesis, and DNA methylation (36), which may contribute to SARS-CoV-2 pathogenesis. Many individuals who recover from COVID continue to have systemic symptoms functionally related to different organs, even though they do not harbor SARS-CoV-2, at least by PCR testing. DNA methylation patterns are thought to be more permanent or persistent, and while the functional contribution of SARS-CoV-2–induced DNA methylation changes to the pathogenicity of the disease is not clear in our study, such epigenetic changes potentially occurring in humans with COVID-19 could lead to symptoms from persistent changes in dysregulated gene expression in infected tissues, even in the absence of tissue viral burden.

In summary, we present a murine model of SARS-CoV-2–induced, rapidly developing systemic toxicity that involves multiple organs, causes severe morbidity and near organismal demise, and recapitulates many clinical phenotypes that are associated with a poor prognosis in humans with COVID-19. Our observations shed insight on the role of metabolic and epigenetic reprogramming in the pathogenesis of multiorgan involvement and lethality in COVID-19.

## Methods

Supplemental methods are available online with this article.

### Study approval.

All animal experiments were approved by the Animal Research Committee (ARC), as well as the High Containment Use Committee (HCUC) at UCLA and were performed at UCLA biosafety level 3 facility. The accession number for the RNA-seq in this paper is available in NCBI GEO dataset. GEO accession number (GSE162113).

## Author contributions

SL and TY performed bench experiments, and FM completed gene expression analysis. In addition, TY performed cardiac imaging. YW provided human ES cell–derived cardiac muscle. GG performed experiments related to viral infection, splenic cell flow cytometry, and tissue collection. RW, MM, and CF assisted in library preparation and analysis of DNA methylation experiments. YCW performed analysis of splenic cell composition, and ZZ dissected adipose tissue. AP, JH, and TGG performed and analyzed data regarding metabolites and gene expression. VAA and JB did SWAB-Seq testing. GAF examined all histological sections. MP supervised analysis of RNA-seq and DNA methylation data, SR performed electron microscopy, AJL supervised analysis of data regarding splenic cell composition and adipose tissue, and MAT contributed to mitochondrial experiments. All experiments related to SARS-CoV-2 infection, daily monitoring, and tissue harvest were performed and supervised by BD and VA. AD conceptualized the project, supervised all data collection, and wrote the manuscript.

## Supplementary Material

Supplemental data

Supplemental Video 1

Supplemental Video 2

## Figures and Tables

**Table 1:**
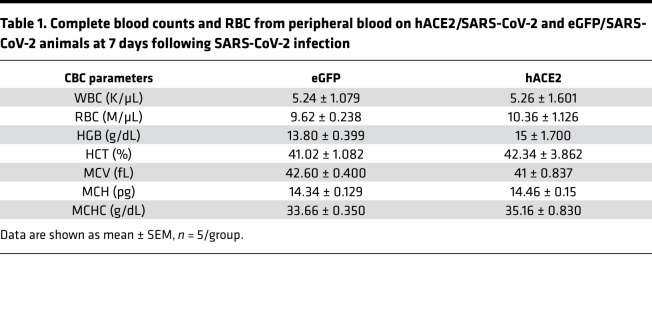
Complete blood counts and RBC from peripheral blood on hACE2/SARS-CoV-2 and eGFP/SARS-CoV-2 animals at 7 days following SARS-CoV-2 infection

**Table 2:**
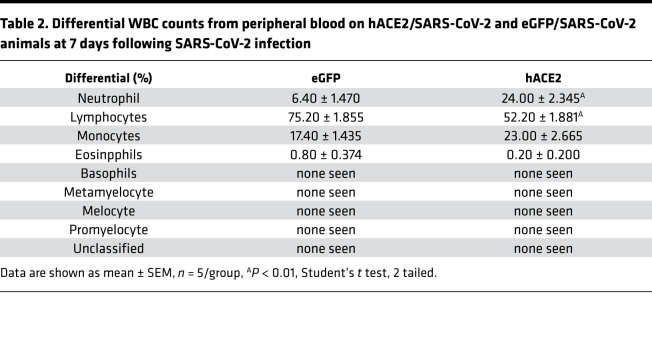
Differential WBC counts from peripheral blood on hACE2/SARS-CoV-2 and eGFP/SARS-CoV-2 animals at 7 days following SARS-CoV-2 infection

**Table 3:**
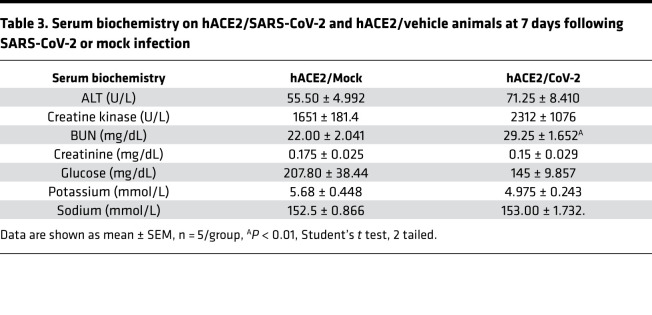
Serum biochemistry on hACE2/SARS-CoV-2 and hACE2/vehicle animals at 7 days following SARS-CoV-2 or mock infection

## Version 1 12/07/2020

In-Press Preview

## Version 2 01/25/2021

Electronic publication
