# Supplementary material for: Metabolic reprogramming and epigenetic changes of vital organs in SARS-CoV-2–induced systemic toxicity
Source: JCI Insight. 2021 Jan 25;6(2):e145027. doi: 10.1172/jci.insight.145027 (PMC7934846; doi:10.1172/jci.insight.145027)
Supplement: Supplemental data [file jciinsight-6-145027-s142.pdf]

Supplementary Materials for

Metabolic reprogramming and epigenetic changes of vital organs in SARS-CoV-2 induced systemic toxicity

Shen Li[#1,2,3,4,5,6], Feiyang Ma[#2,3,4,], Tomohiro Yokota[#1,2,3,4,5,6,], Gustavo Garcia Jr.[7], Amelia Palermo[6,7,11,12], Yijie Wang[1,2,3,4,5,6], Colin Farrell[8], Yu-Chen Wang[1,2,8], Rimao Wu[1,2,3,4,5,6], Zhiqiang Zhou[1,2,8], Calvin Pan[1,2,8], Marco Morselli[3,4,5], Michael A. Teitell[9], Sergey Ryazantsev[6], Gregory A Fishbein[9], Johanna ten Hoeve[6,7,11,12], Valerie A. Arboleda[8,9], Joshua Bloom[8,13,14], Barbara Dillon[10], Matteo Pellegrini[3,4,5], Aldons J Lusis[1,2,8,], Thomas G Graeber[4,6,7,11,12], Vaithilingaraja Arumugaswami*[4,7], Arjun Deb*[1,2,3,4,5,6]

This PDF file includes:

Materials and Methods
Supplemental Figures 1 to 6 with accompanying figure legends
Legends for Supplemental Movies 1A to 1B
Legends for Supplemental Table 1 to 4

Other Supplementary Materials for this manuscript include the following:

Supplemental Movies 1A to 1B
Supplemental Table 1 to 4

Materials and Methods

Animal care and use

All animal studies were approved by the Animal Research Committee, University of California, Los Angeles. Male C57Bl/6 mice (000664, Jackson Labs) (14-17 weeks) were housed in groups and fed standard chow diets. AAV9-CMV-hACE2 (AAV-200183, Vector Biolabs) or control AAV9-CMV-eGFP viruses were purchased from Vector Biolabs. Animals were injected intravenously with 100-µl injection containing $2x10^{12}$ genomic copies of AAV-CMV-hACE2 or control (AAV-CMV-eGFP). Animals were housed in BSL-3 facility for the duration of the experiment (n=5/cage). Cage food weight and individual mouse body weight were recorded daily after SARS-CoV-2 virus infection.

Biosafety

All aspects of this study involving live SARS-CoV-2 virus were approved by the University of California, Los Angeles Institutional Biosafety Committee (IBC). All work with SARS-CoV-2 was performed utilizing approved Biosafety Level 3 (BSL3) standard operating procedures and was conducted in our UCLA performance-validated BSL3 laboratory, designed adhering to the guidelines recommended by the Biosafety in Microbiological and Biomedical Laboratories (BMBL), the U.S. Department of Health and Human Services, the Los Angeles Department of Public Health (LADPH) and the Centers for Disease Control and Prevention (CDC).

SARS-CoV2 infection

SARS-CoV-2, isolate USA-WA1/2020, was got from the Biodefense and Emerging Infections (BEI) Resources of the National Institute of Allergy and Infectious Diseases (NIAID). SARS-CoV-2 was passaged once in Vero-E6 cells (ATCC) and viral stocks were aliquoted and stored at -80 C. Virus titer was determined by plaque assay using Vero E6 Cells. 200µL of SARS-CoV2 (0.5×10^6 PFU/mL) was injected intraperitoneally.

Echocardiography

Echocardiography was performed prior to SARS-CoV-2 infection and at 1,4 and 7 days post SARS-COV-2 injection. For echocardiography, animals were anesthetized with a mixture of isoflurane and 95% O2. Vevo 3100LT imaging system and a 30-mHz transducer (MX400, VisualSonics) were used to acquire short/long axis B-mode and M-mode images. All measurements and calculations were conducted using Vevo Lab software.

Electrocardiography

Electrocardiography was performed on animals following anesthesia with 0.7% isoflurane administered through a nose cone. Animal body temperature was maintained using a heat pad. The electrocardiogram was recorded from 3 micro-hook Electrodes connected to the right forelimb and each hindlimb (limb leads). The signal was acquired for about 3 minutes using Bio Amp (AD instruments). The recorded signals were analyzed by LabChart software (AD instruments).

Non-invasive blood pressure measurement.

Blood pressure was measured using a CODA noninvasive blood pressure monitoring system (CODA^{TM} Monitor, Kent Scientific). The animal was anesthetized with 0.7%

isoflurane administered through a nose cone prior to recording of blood pressure. The animals were placed on a heat pad with warming cover to maintain tail temperature around 35°C. Multiple recordings were obtained for a duration of 10m minutes using a tail cuff sensor.

Histology

Animals were euthanized before harvest and fixation of tissues. Tissues were fixed in 4% paraformaldehyde and subsequently subjected to paraffin or frozen section preparation. Paraffin embedded tissues were sectioned at 5μm thickness, and stained with hematoxylin and eosin or Phosphotungstic acid-hematoxylin (PTAH) staining. Tissues for frozen section were embedded in Tissue-Tek O.C.T and sectioned at 10μm thickness for immunostaining or Oil Red O staining. For immunostaining, tissue sections were post fixed in 4% paraformaldehyde for 15 min and permeabilized in 0.1% triton X-100 for 15 min. Sections were then blocked in 10% species-specific normal serum in 1% BSA/PBS for 1 h, and primary antibodies diluted in 1% BSA/PBS at 4˚C overnight. Secondary antibodies were diluted in PBS and incubated with the sections for 1 h. Samples were counterstained with DAPI (D3571, Invitrogen) and mounted with SlowFade Gold Antifade reagent (S36936, Invitrogen). Images were taken using Nikon Eclipse Ti2 microscopy (Nikon,USA) with DS-Ri2 brightfield camera or C2+ confocal system.

Antibodies and reagents

SARS-CoV-2 (2019-nCoV) Spike S1 antibody (40150-R007, Sino Biological); Cardiac Troponin I antibody (ab47003, Abcam); CD45 (ab1416, Abcam); CD68 (ab125212, Abcam); Alexa Flour 594 conjugated WGA (W11262, Invitrogen).

Mass cytometry analysis of Spleen

To characterize splenic cell populations we used mass cytometry analysis. Maxpar Cell Surface Staining protocol was used as per the manufacturer recommendations (Fluidigm) along with minor modifications[1] and shown in other recent studies[2]. Splenic cells were isolated to assess immunological responses from uninfected (mock), eGFP/SARS-CoV-2, and hACE2/SARS-CoV-2 infected conditions following intra-peritoneally injections. The spleen was briefly incubated in cold 1x PBSand mechanically disrupted with the bottom of a syringe. Using sterile 70-μm cell strainers (Falcon cell strainer, Fisher Scientific), homogenates were filtered through with 10 ml of cold 1X PBS. Red blood cells were lysed by using ACK Lysing Buffer (GIBCO) for about 2 minutes at room temperature. Cell were then resuspended in 8 ml of 1X PBS to neutralize ACK Lysing Buffer. Approximately 10 x 10^6 cells were aliquoted into a 1.5 mL screw cap tube after counting. Cells were centrifuged between steps at 300 g for 5 mins. Cells were then resuspended in 500 μl of 1X PBS with Cell-ID Cisplatin (Fluidigm) for 5 minutes. Cells were centrifuged andresuspended in 50 μl of Maxpar Staining Buffer with FC Blocker solution (0.5 - 1 μg of CD16/CD32, clone: 93, eBioscience), cells were incubated for 10 minutes. Subsequently, 50 μl of metal-conjugated antibody cocktail (with optimal concentrations), as listed in Table X, were added. Cells incubated with antibody cocktail for 30 mins at room temperature. The cells were then washed two times with Maxpar Staining Buffer before adding the cell intercalation solution (Maxpar Fix and Perm Buffer; Cell-ID Intercalator-Ir) with an overnight incubation at 4° C. Cells were then washed with Maxpar Staining Buffer with new centrifugation speed of 800g for 5 minutes, resuspended in water, and subjected to mass cytometry. Individual cells are ionized and analyzed by Helios CyTOF Mass Cytometer. Cell subpopulations were

analyzed using the CyTOF Software v6.7 (Fluidigm) and FlowJo software program (FlowJo, LLC).

	**Metal Conjugate**	**Target Epitope**	**Source**
1	89Y	CD45	Fluidigm
2	139La	CD27	Fluidigm
3	141Pr	Ly-6G	Fluidigm
4	142Nd	CD11c	Fluidigm
5	143Nd	CD69	Fluidigm
6	145Nd	CD4	Fluidigm
7	146Nd	F4/80	Fluidigm
8	147Sm	CD317	Fisher (eBioscience)
9	148Nd	CD11b (Mac-1)	Fluidigm
10	149Sm	CD19	Fluidigm
11	150Nd	Ly-6C	Fluidigm
12	152Sm	CD3e	Fluidigm
13	153Eu	CD274 (PD-L1)	Biolegend
14	159Tb	CD279(PD1)	Biolegend
15	160Gd	CD62L (L-selectin)	Fluidigm
16	161Dy	CD40	Fluidigm
17	162Dy	CD366 (Tim3)	Fluidigm
18	163Dy	CD138	Fluidigm
19	164Dy	CD197/CCR7	Fluidigm
20	167Er	CD335 (NKp46)	Fluidigm
21	168Er	CD8a	Fluidigm
22	170Er	CD80 (B7-1)	Biolegend
23	172Yb	CD86	Fluidigm
24	173Yb	CD44	Fluidigm
25	174Yb	I-A/I-E (MHC class II)	Fluidigm
26	175Lu	CD103	Fluidigm
27	176Yb	CD45R (B220)	Fluidigm

Complete blood count and Blood biochemistry

Peripheral blood was obtained via cardiac puncture. Whole blood or serum was collected in heparinized tubes (365965, BD Biosciences) or in serum tubes (365963, BD Biuosciences). 150μL whole blood was used for determining complete/differential blood counts and 130μL serum was used for biochemistry analysis. Both Complete blood count and serum biochemistry were tested by IDEXX Bioanalytic (IDEXX).

Determination of Serum metabolites

For metabolic profiling of serum, 50μL of serum was mixed with 50μLH_2O and 400μL methanol, the sample vortexed vortex and kept at -80°C for 20 minutes. Samples were centrifuged and the cell free supernatant mixed with 300μl H_2O and 400μl chloroform. Aqueous phase was harvest and dried by vacuum evaporator. Dried metabolites were resuspended in 50% ACN:water and 1/10th was loaded onto a Luna 3um NH2 100A (150 × 2.0 mm) column (Phenomenex). The chromatographic

separation was performed on a Vanquish Flex (Thermo Scientific) with mobile phases A (5 mM NH4AcO pH 9.9) and B (ACN) and a flow rate of 200 μl/min. A linear gradient from 15% A to 95% A over 18 min was followed by 9 min isocratic flow at 95% A and reequilibration to 15% A. Metabolites were detection with a Thermo Scientific Q Exactive mass spectrometer run with polarity switching (+3.5 kV/− 3.5 kV) in full scan mode at 70000 resolution with an m/z range of 65-975. Maven was used to quantify the targeted metabolites by area under the curve using expected retention time and accurate mass measurements (< 5 ppm).

Q-PCR

All samples were stored in RNAlater stabilization solution and subsequently total RNA was extracted using RNA mini Kit (7326830, BioRad). cDNA was synthesized by using iScript cDNA Synthesis Kit (1708890, BioRad) and qPCR performed.

Library construction and sequencing

For RNA-sequencing, libraries were prepared by the Technology Center for Genomics & Bioinformatics at UCLA using Illumina TruSeq Stranded Total RNA Sample Prep kit and sequenced with 50bp single end reads on an Illumina HiSeq3000.

Analysis of RNA sequencing data

The reads were mapped with STAR 2.5.3a[3] to the human genome (hg38) for the cultured human cell libraries or mouse genome (mm10) for the mouse cell libraries. The counts for each gene were obtained using --quantMode GeneCounts in STAR commands, and the other parameters during alignment were set to default. Differential expression analyses were carried out using DESeq2[4]. Single variable comparison was carried out for each tissue type. For the comparison with multiple tissue types, the tissue type was considered a second variable to build the model, and only the effects related to the different treatments were extracted. Counts normalized by sequencing depth were obtained using DESeq2 estimate size Factors function with default parameters. Principle component analysis was performed on the log10 normalized counts using the R function prcomp. Genes with adjusted p value < 0.05 were considered significantly differentially expressed. Significantly up-regulated or down-regulated genes were uploaded to the Enrichr for the pathway analyses[5]. GO biological pathways and KEGG pathways were combined and ranked by p value, and the top 10 pathways with the lowest p values were plotted. The accession number for the RNA sequencing in this paper is available in NCBI GEO dataset. GEO accession number (GSE162113).

DNA methylation

Heart and Kidney genomic DNA were extracted by DNA purification Kit (80204, Qiagen). RRBS libraries were aligned to the mouse reference genome (mm10) and methylation values called for using BSBolt (v1.3.4). Methylation values were called for CpG with a minimum of five reads covering the sites with a mapping quality >= 20 and a base call quality >= 20. We used a logistic regression model with overdispersion correction implemented in MethylKit (v1.14.2) to identify DMS associated with hACE2 knockout[6,7]. The SLIM method was used to adjust p-values to q-values[8]. Sites with a q-value less than 0.01 were reported as differentially methylated sites (DMS). DMS were annotated by proximity to the nearest TSS using genomation (v1.20.0) [6,7].

Transmission electron microscopy

Transmission electron microscopy was performed as recently described[9]. The heart was harvested immediately after euthanasia, fixed in 2% glutaraldehyde of PBS at 4°C for 3 hour. Fixed samples were embedded in low-viscosity resin (Agar, UK) in the following manner: osmicated with 1% OsO4; stained with 3% uranyl-acetate; dehydrated in 30-50-70-95-100% ethanol and embedded in low-viscosity resin (Agar, UK). Plastic-embedded samples were sectioned using UCT ultramicrotome (Leica, Austria) and diamond knife (Diatome, Austria). 50-55nm thickness section were mounted on home-made EM grid(s) with plastic-carbon support film, stained with saturated uranyl-acetate and Sato's lead-citrate. Sections were imaged using JEM1200EX transmission electron microscope (JEOL, Japan) at 80 kV equipped with BioScan600W digital camera (Gatan, USA).

hPSC derived cardiomyocyte differentiation and SARS-CoV2 infection

We derived cardiomyocyte (CM) from hESC line H9 using the previously described method[10]. Briefly, hPSCs were maintained in mTeSR1 (stemcell technology). RPMI 1640 supplemented with B27 minus insulin (Invitrogen) was used as differentiation medium. On day 0-1, 6 µM CHIR99021 (selleckchem) was added into differentiation medium. Between day 3-5, 5 µM IWR1 (Sigma-Aldrich) was added to refreshed differentiation medium. After day 7 medium was replaced with RPMI 1640 plus B27 CM maintain medium. From day 10-11, RPMI 1640 without D-glucose supplemented with B27 was transiently used for metabolic purification of CMs[11].

For hiPSC-CM infection, viral inoculum (MOI of 0.1) was prepared using serum-free media (250 µl/well) and was added directly to the cells. After 1 hour of absorption (37°C, 5% CO_2), the inoculum was replaced with fresh culture media (500 µl/well). For mock infection, culture media alone was used. The cells were incubated at 37°C with 5% CO_2 for 24 to 48 hours. At the desired endpoints, the cells were fixed with 4% paraformaldehyde (for immunocytochemistry), and 80% methanol (metabolomics studies).

Statistics

All data is presented as mean ± standard error (S.E.M.) and mentioned in the figure legends. Statistical analysis was performed using GraphPad (Prism) software using Student's t-test (Two tailed) and repeated measures two-way ANOVA with Sidak's multiple comparisons analysis as appropriate. A p value <0.05 was considered statistically significant.

References

1 Chiang, N., de la Rosa, X., Libreros, S. & Serhan, C. N. Novel Resolvin D2 Receptor Axis in Infectious Inflammation. *J Immunol* **198**, 842-851, doi:10.4049/jimmunol.1601650 (2017).

2 Martinez, L. E., Garcia, G., Jr., Contreras, D., Gong, D., Sun, R. & Arumugaswami, V. Zika Virus Mucosal Infection Provides Protective Immunity. *J Virol* **94**, doi:10.1128/JVI.00067-20 (2020).

3 Dobin, A., Davis, C. A., Schlesinger, F., Drenkow, J., Zaleski, C., Jha, S. *et al.* STAR: ultrafast universal RNA-seq aligner. *Bioinformatics* **29**, 15-21, doi:10.1093/bioinformatics/bts635 (2013).

4 Love, M. I., Huber, W. & Anders, S. Moderated estimation of fold change and dispersion for RNA-seq data with DESeq2. *Genome Biol* **15**, 550, doi:10.1186/s13059-014-0550-8 (2014).

5 Kuleshov, M. V., Jones, M. R., Rouillard, A. D., Fernandez, N. F., Duan, Q., Wang, Z. *et al.* Enrichr: a comprehensive gene set enrichment analysis web server 2016 update. *Nucleic Acids Res* **44**, W90-97, doi:10.1093/nar/gkw377 (2016).

6 Akalin, A., Kormaksson, M., Li, S., Garrett-Bakelman, F. E., Figueroa, M. E., Melnick, A. *et al.* methylKit: a comprehensive R package for the analysis of genome-wide DNA methylation profiles. *Genome Biology* **13**, R87, doi:10.1186/gb-2012-13-10-r87 (2012).

7 Akalin, A., Franke, V., Vlahovicek, K., Mason, C. E. & Schubeler, D. Genomation: a toolkit to summarize, annotate and visualize genomic intervals. *Bioinformatics* **31**, 1127-1129, doi:10.1093/bioinformatics/btu775 (2015).

8 Wang, H. Q., Tuominen, L. K. & Tsai, C. J. SLIM: a sliding linear model for estimating the proportion of true null hypotheses in datasets with dependence structures. *Bioinformatics* **27**, 225-231, doi:10.1093/bioinformatics/btq650 (2011).

9 Yokota, T., McCourt, J., Ma, F., Ren, S., Li, S., Kim, T. H. *et al.* Type V Collagen in Scar Tissue Regulates the Size of Scar after Heart Injury. *Cell*, doi:10.1016/j.cell.2020.06.030 (2020).

10 Lian, X., Hsiao, C., Wilson, G., Zhu, K., Hazeltine, L. B., Azarin, S. M. *et al.* Robust cardiomyocyte differentiation from human pluripotent stem cells via temporal modulation of canonical Wnt signaling. *Proc Natl Acad Sci U S A* **109**, E1848-1857, doi:10.1073/pnas.1200250109 (2012).

11 Sharma, A., Li, G., Rajarajan, K., Hamaguchi, R., Burridge, P. W. & Wu, S. M. Derivation of highly purified cardiomyocytes from human induced pluripotent stem cells using small molecule-modulated differentiation and subsequent glucose starvation. *J Vis Exp*, doi:10.3791/52628 (2015).

Supplemental Figure 1

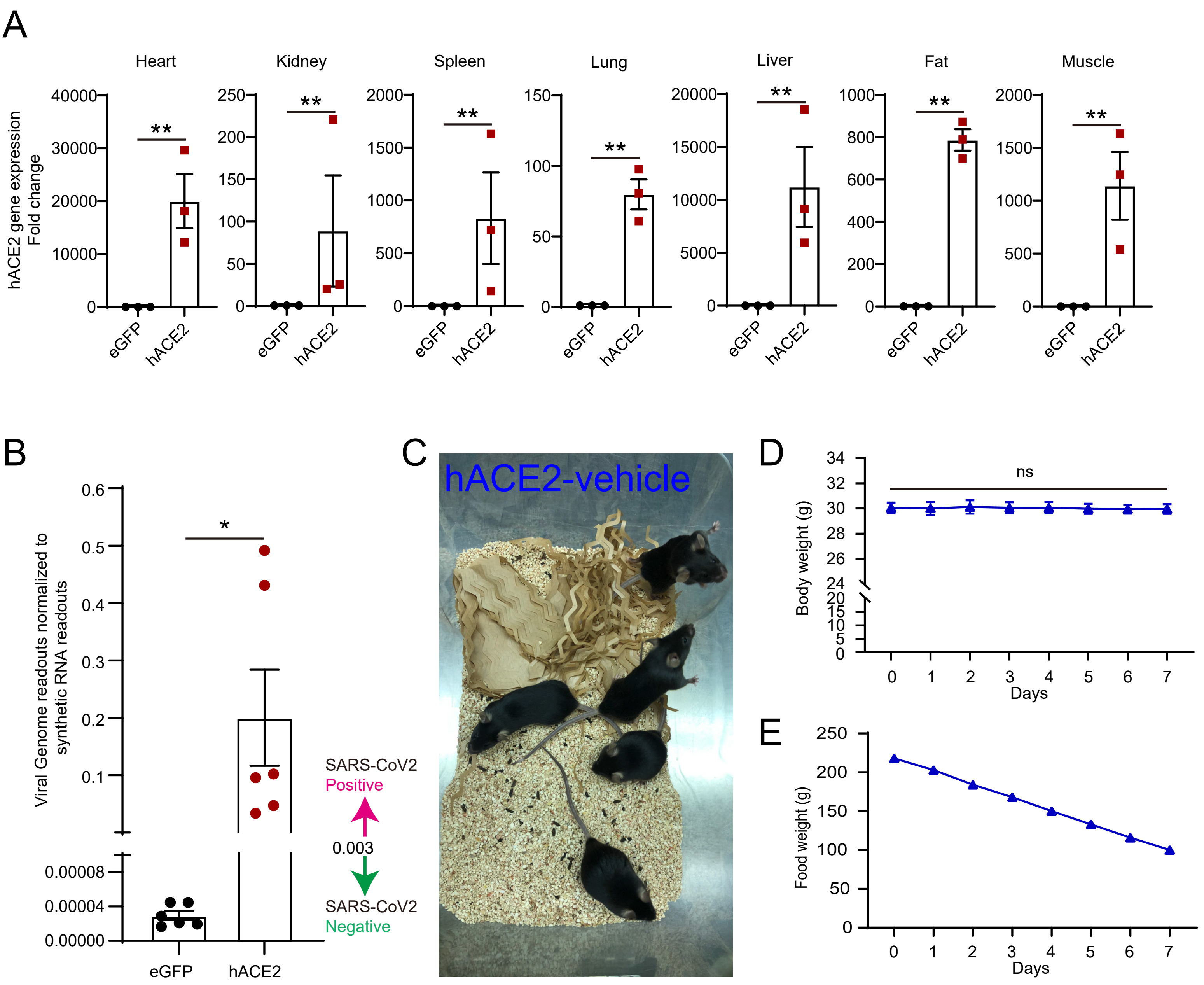

Supplemental Figure 1. Expression of hACE2 in multiple organs following AAV-9 eGFP or AAV-9 hACE2 injection and body weight and food intake in hACE2/vehicle control animals. (A) qPCR comparing hACE2 expression in multiple organs between AAV-9 hACE2 and AAV-9 eGFP group two weeks following AAV-9 injection (data shown as mean±S.E.M., n=3, **p<0.01 for all organs, Student's t-test, 2 tailed). **(B)** Swab seq to determine viral genome copies using next generation sequencing. Synthetic viral RNA is used as a positive control and ratio of true viral reads to synthetic viral reads of >0.003 is considered to be positive for infection (data shown as mean±S.E.M., n=3, *p<0.05, Student's t-test, 2 tailed). **(C-E)** hACE2/vehicle group animals did not demonstrate any features of **(C)** morbidity or inactivity or **(D)** weight loss or **(E)** decreased food consumption (n=5) (ns:not significant)

Supplemental Figure 2

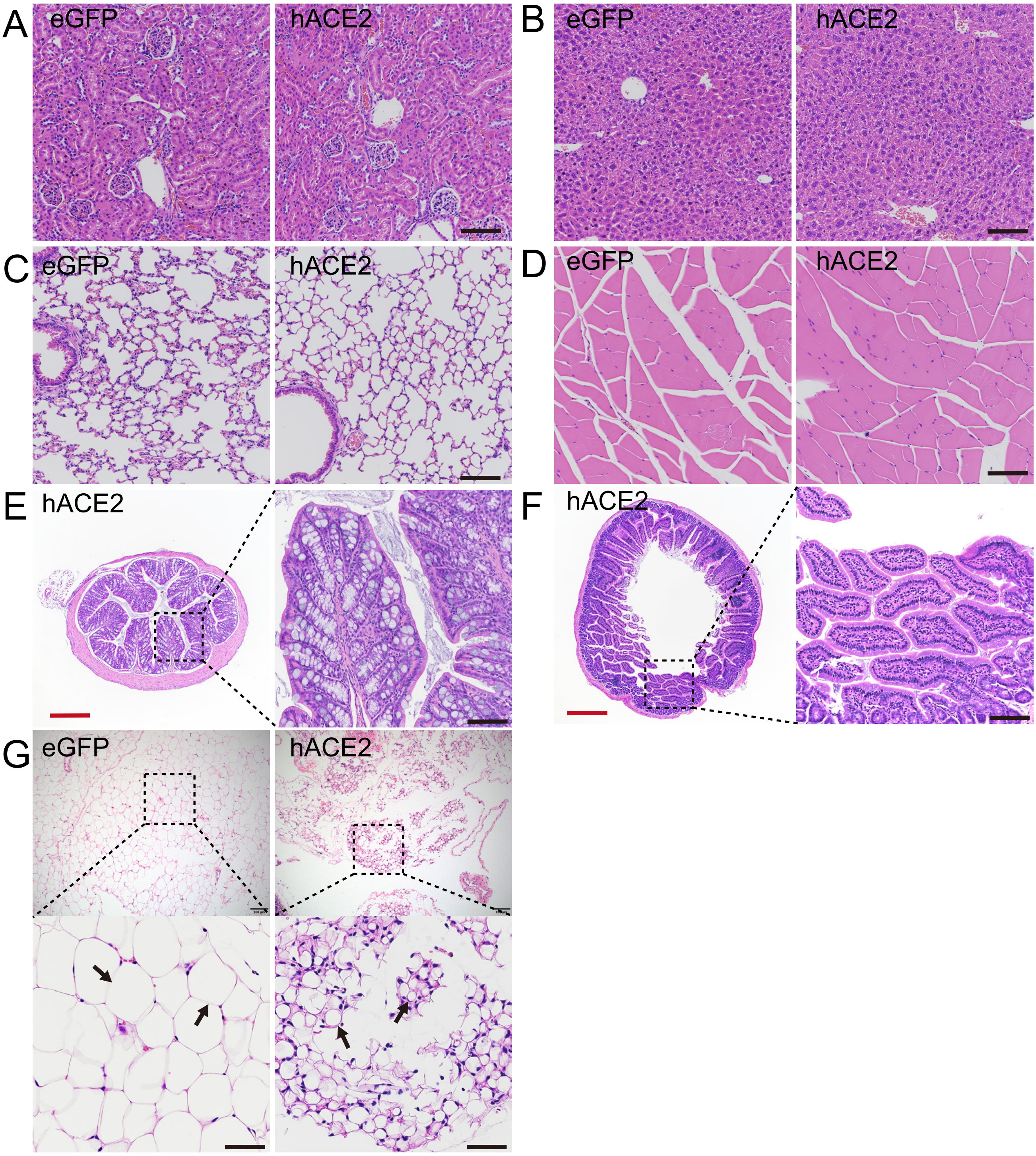

Supplemental Figure 2. Histology of vital organs in murine model of SARS-CoV-2 induced systemic toxicity. Hematoxylin/eosin staining of **(A)** kidney **(B)** liver **(C)** lung and **(D)** skeletal muscle of eGFP/SARS-CoV-2 and hACE2/SARS-CoV-2 group (n=5/group) and **(E,F)** normal histology of **(E)** small and **(F)** large intestine in hACE2/SARS-CoV-2 animals (n=4). Red scale bar: 500 µm; black scale bar: 100 µM. **(G)** Hematoxylin/eosin staining demonstrating decreased adipocyte size in hACE2/SARS-CoV-2 animals (arrows); lower panel shows magnified view of highlighted rectangle. Scale bar: 50 µm.

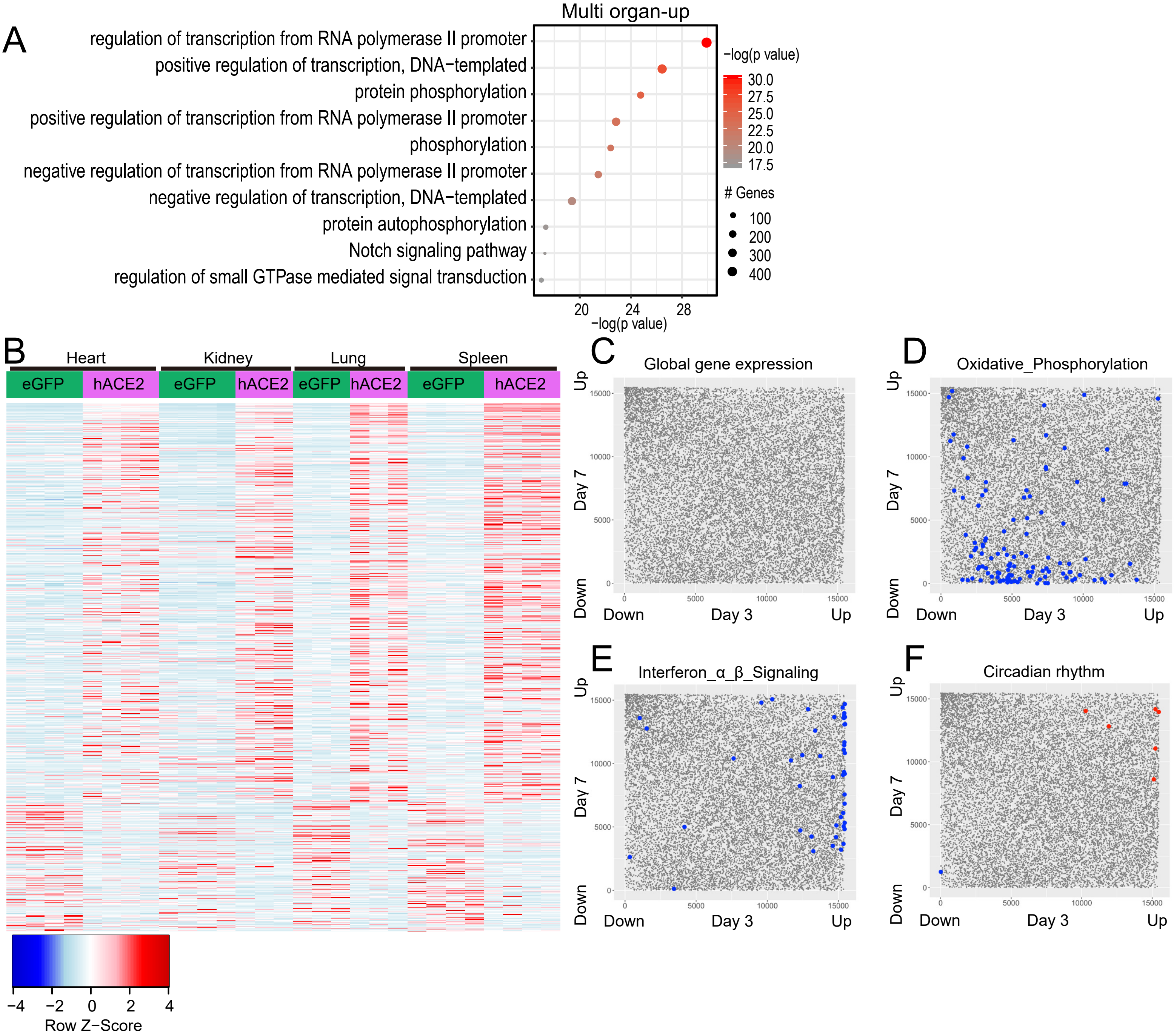

Supplemental Figure 3. Patterns of gene expression changes in vital organs of eGFP/SARS-CoV-2 and hACE2/SARS-CoV-2 animals at 7 days post SARS-CoV-2 infection. (A) Gene ontology analysis demonstrating pathways enriched by genes that are commonly upregulated in the heart, kidney, lung and spleen of hACE2/SARS-CoV-2 animals compared to eGFP/SARS-CoV-2 animals. **(B)** Heat map of differentially expressed genes across 4 organs demonstrating similar patterns of up and downregulation across all 4 organs in the hACE2/SARS-CoV-2 animals versus the eGFP/SARS-CoV-2 group. (Heart: n=4/eGFP, n=4/hACE2; Kidney: n=4/eGFP, n=3/hACE2; Lung: n=3/eGFP, n=3/hACE2; Kidney: n=4/eGFP, n=4/hACE2) **(C)** Genes were ordered by their average differential expression p-value-based rank in day 3 tissues (heart, lung, kidney, liver, muscle, fat) versus day 7 tissues (heart, lung, kidney, spleen). A lack of correlation is likewise observed when only the paired-tissues from day 3 and day 6 are analyzed. **(D-F)** Distinct patterns of gene expression of **(D)** oxidative phosphorylation genes and **(E)** Interferon signaling genes between Day 3 and Day 7 tissues following SARS-CoV-2 infection. **(F)** Circadian clock genes (red) (Ciart, Cipc, Per1, Per2, Timeless) were upregulated at both time points while clock gene (blue) was downregulated at both time points (p=4.1e-05)

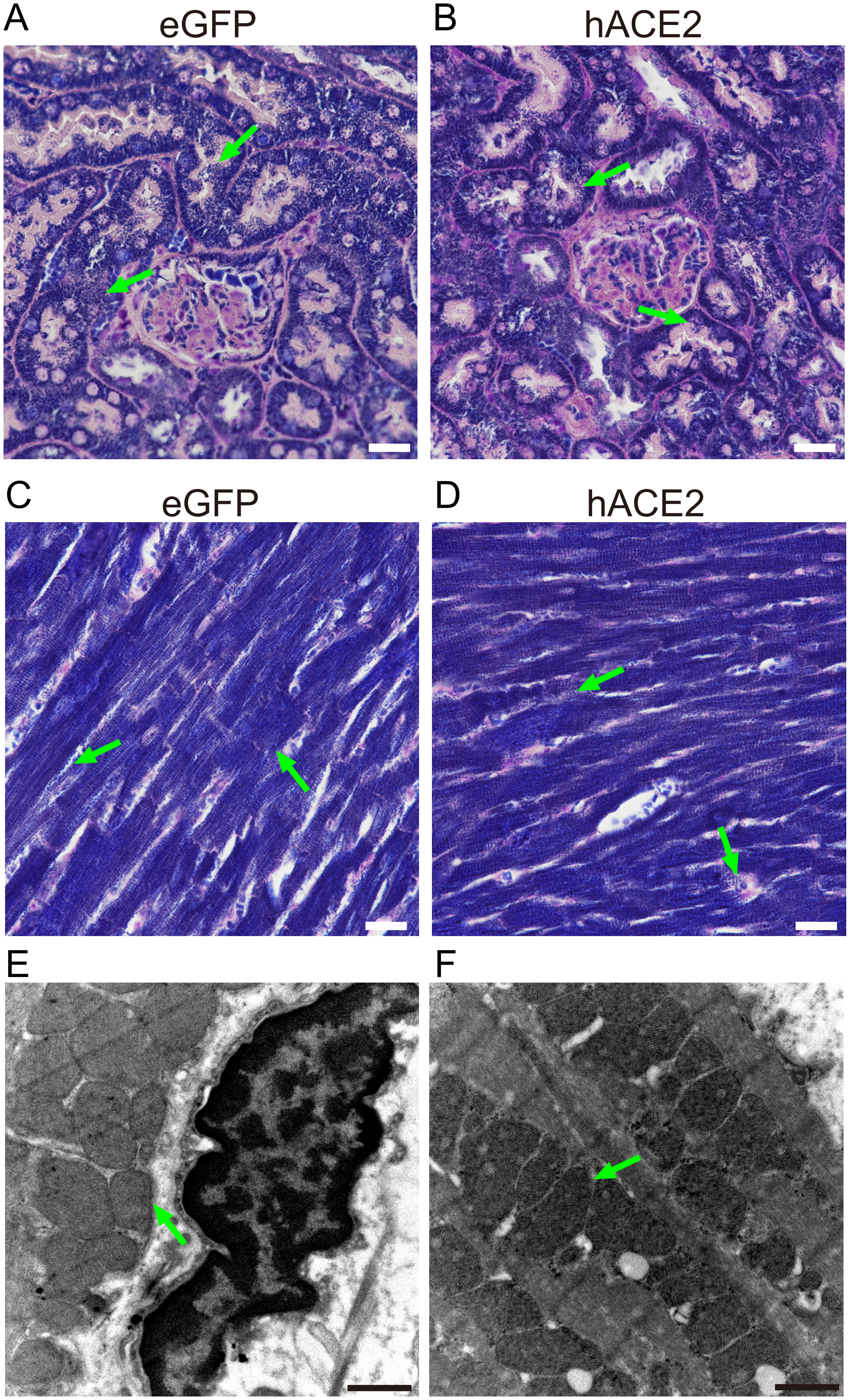

Supplemental Figure 4. Mitochondrial number and morphology are not significantly different in tissues of hACE2/SARS-CoV-2 animals compared to eGFP/SARS-CoV-2 animals at 7 days following SAS-CoV-2 infection. (A,B) Phosphotungstic acid-hematoxylin (PTAH) stain of kidneys demonstrates no difference in the number of mitochondria between **(A)** eGFP/SARS-CoV-2 and **(B)** hACE2/SARS-CoV-2 groups. (n=3/group, arrows). Scale bar: 20 µm. **(C,D)** Phosphotungstic acid-hematoxylin (PTAH) stain of heart demonstrates no difference in the number of mitochondria in cardiomyocytes between **(C)** eGFP/SARS-CoV-2 and **(D)** hACE2/SARS-CoV-2 groups. (n=3/group, arrows). Scale bar: 20 µm. **(E,F)** Transmission electron microscopy of the heart demonstrates normal arrangement and morphology of the mitochondria in cardiomyocytes of **(E)** eGFP/SARS-CoV-2 and **(F)** hACE2/SARS-CoV-2 groups (n=2/group, arrows). Scale bar: 1 µm.

Supplemental Figure 5

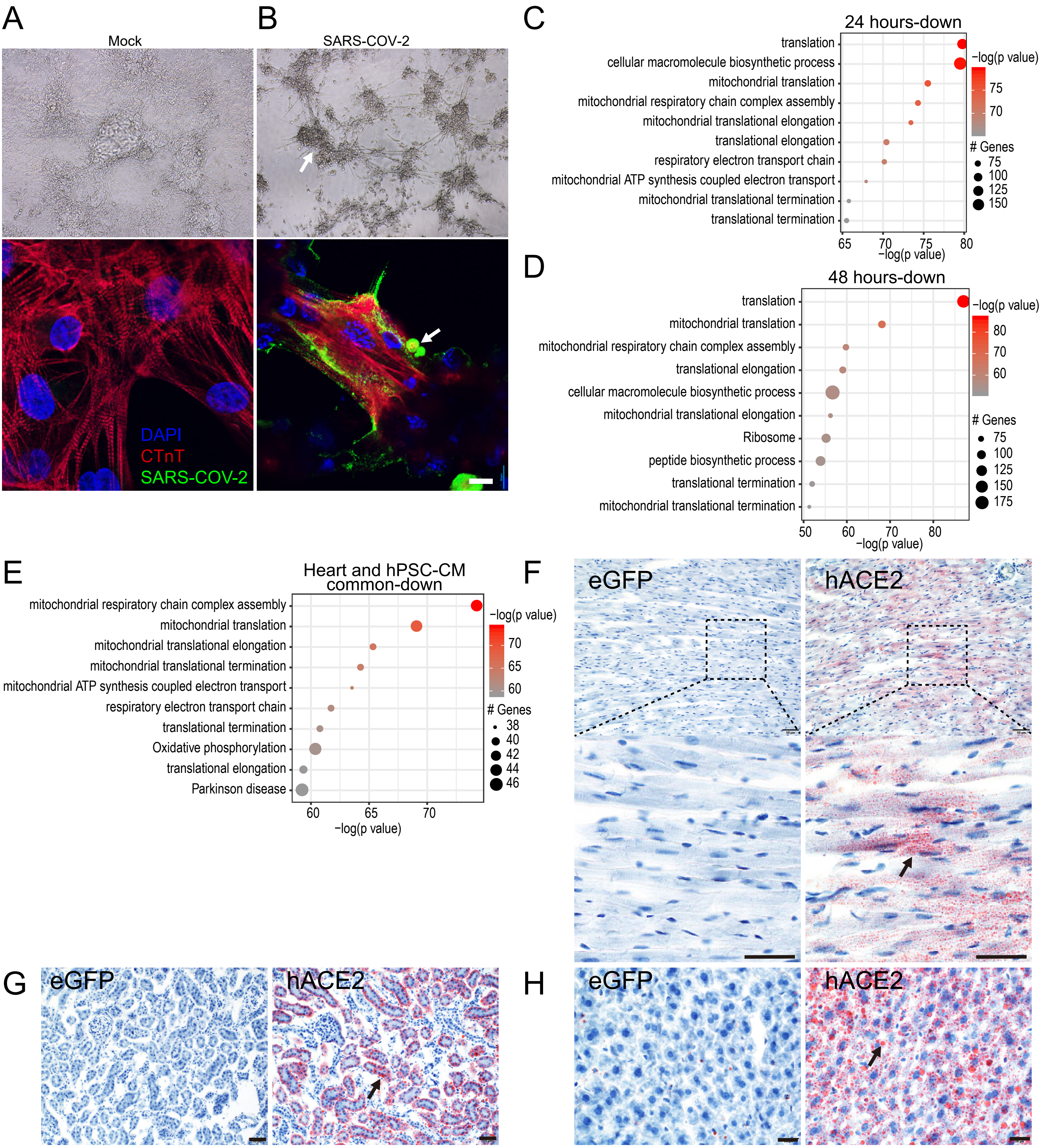

Supplemental Figure 5. Phenotype and gene expression changes in hPSC derived cardiomyocytes infected with SARS-CoV-2 and lipid deposition in vital organs of hACE2/SARS-CoV-2 animals. (A,B) Human embryonic stem cell derived cardiomyocytes **(A)** mock infected or **(B)** infected with SARS-CoV-2 and then imaged 48 hours later demonstrates rounding up and clumping of SARS-CoV-2 infected cells (arrow). **(A,Blower panel) I**mmunostaining for cardiac troponin and viral S protein demonstrating expression of viral protein (arrow) within human cardiomyocytes in SARS-CoV-2 infected cells but not in mock infected cells. Scale bar: 10 µm. **(C,D)** Gene ontology analysis demonstrating pathways enriched by downregulated genes in human cardiomyocytes at **(C)** 24 hours (n=3 for mock, n=3 for SARS-COV-2) and **(D)** 48 hours (n=3 for mock, n=3 for SARS-COV-2)following infection with SARS-CoV-2. **(E)** Gene ontology analysis demonstrating pathways enriched by genes differentially downregulated in hPSC derived cardiomyocytes (24 and 48 hours following infection) and hearts of hACE2/SARS-CoV-2 animals 7 days following SARS-CoV-2 infection in vivo. **(F-H)** Oil Red O staining of sections of **(F)** hearts of eGFP and hACE2 animals 7 days after SARS-CoV-2 infection and lower panel in higher magnification demonstrating lipid deposits (arrow). Scale bar: 50 µm. **(G)** Kidney and **(H)** liver demonstrating abundant lipid deposition (arrows) in hACE2/SARS-CoV-2 but not in eGFP/SARS-CoV-2 group. Scale bar: 50 µm in **(G)** and 20 µm in **(H)**.

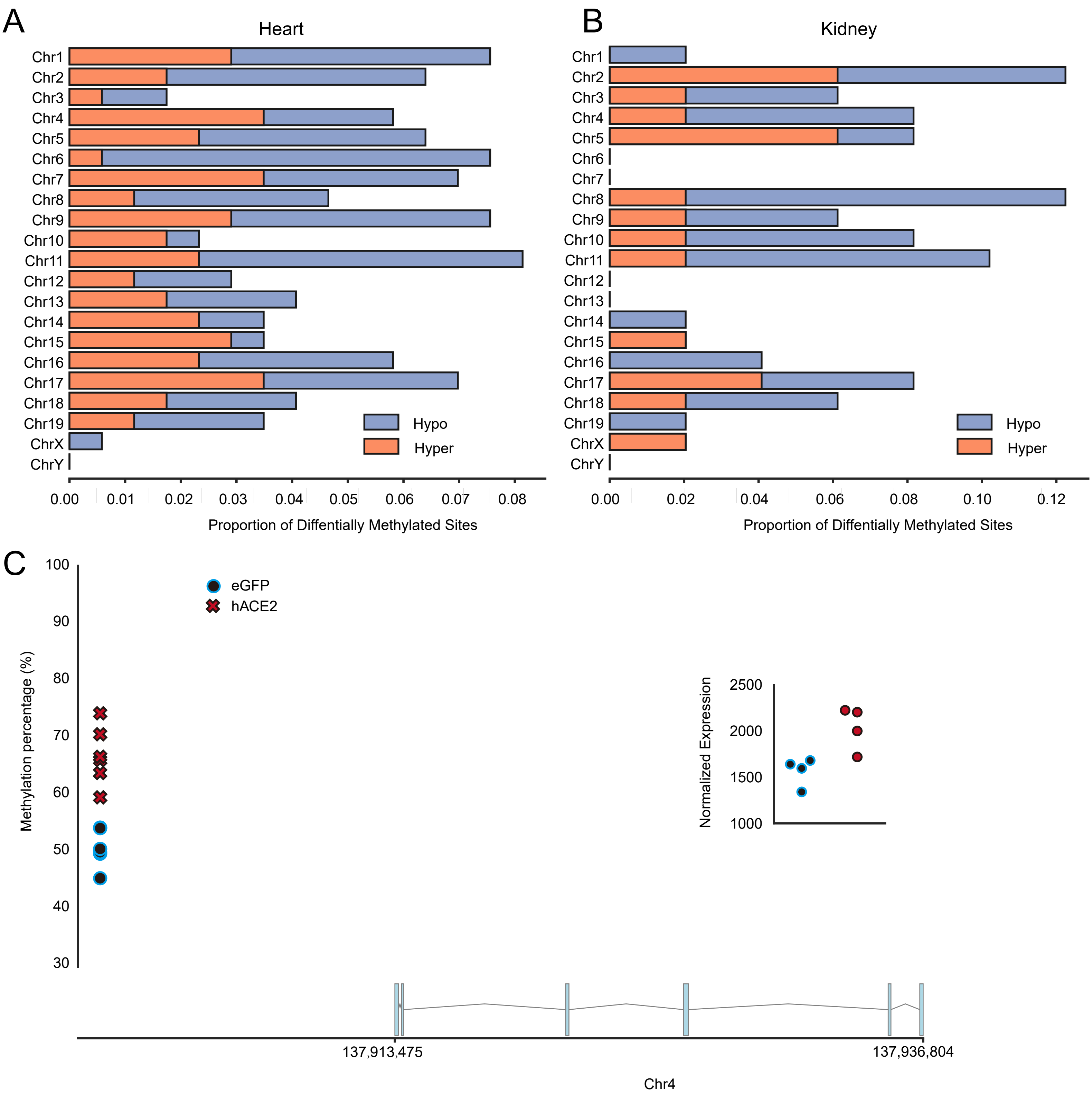

Supplemental Figure 6. SARS-CoV-2 induces changes in DNA methylation in internal organs. (A,B) Distribution of differentially methylated sites across the genomes of **(A)** heart and **(B)** kidney of hACE2/SARS-CoV-2 animals compared to eGFP/SARS-CoV-2 animals shown according to the location of the site in the chromosome (n=3/group). **(C)** Percentage of differentially methylated sites approximately 13kB distant from the transcription start site of the Ece1 gene and corresponding change in gene expression of Ece1 in the heart. (n=3/group).

Supplemental Movies 1A.

Video clip demonstrating activity of eGFP/SARS-CoV-2 group at 7 days following SARS-CoV-2 infection.

Supplemental Movies 1B.
Video clip demonstrating activity of hACE2/SARS-CoV-2 group at 7 days following SARS-CoV-2 infection

Supplemental Table 1.
Gene expression changes in tissues of eGFP/SARS-CoV-2 and hACE2/SARS-CoV-2 animals at 3 days following SARS-CoV-2 infection (only differentially expressed genes shown)

Supplemental Table 2.
Gene expression changes in tissues of eGFP/SARS-CoV-2 and hACE2/SARS-CoV-2 animals at 7 days following SARS-CoV-2 infection (only differentially expressed genes shown)

Supplemental Table 3.
Gene expression changes in hESC derived cardiomyocytes at 24 and 48 hours after SARS-CoV-2 infectionb in vitro.

Supplemental Table 4.
Differentially methylated sites in heart and kidney of hACE2/SARS-CoV-2 animal versus eGFP/SARS-CoV-2 animals at 7 days following SARS-CoV-2 infection.
